# STAT1 is essential for HSC function and maintains MHCII^hi^ stem cells that resist myeloablation and neoplastic expansion

**DOI:** 10.1182/blood.2021014009

**Published:** 2022-10-06

**Authors:** Juan Li, Matthew J. Williams, Hyun Jung Park, Hugo P. Bastos, Xiaonan Wang, Daniel Prins, Nicola K. Wilson, Carys Johnson, Kendig Sham, Michelle Wantoch, Sam Watcham, Sarah J. Kinston, Dean C. Pask, Tina L. Hamilton, Rachel Sneade, Amie K. Waller, Cedric Ghevaert, George S. Vassiliou, Elisa Laurenti, David G. Kent, Berthold Göttgens, Anthony R. Green

**Affiliations:** 1 Wellcome–Medical Research Council Cambridge Stem Cell Institute, Jeffrey Cheah Biomedical Centre; 2 Department of Haematology, University of Cambridge, Cambridge, United Kingdom; 3 Department of Biology, University of York, York, United Kingdom

## Abstract

Adult hematopoietic stem cells (HSCs) are predominantly quiescent and can be activated in response to acute stress such as infection or cytotoxic insults. STAT1 is a pivotal downstream mediator of interferon (IFN) signaling and is required for IFN-induced HSC proliferation, but little is known about the role of STAT1 in regulating homeostatic hematopoietic stem/progenitor cells (HSPCs). Here, we show that loss of STAT1 altered the steady state HSPC landscape, impaired HSC function in transplantation assays, delayed blood cell regeneration following myeloablation, and disrupted molecular programs that protect HSCs, including control of quiescence. Our results also reveal STAT1-dependent functional HSC heterogeneity. A previously unrecognized subset of homeostatic HSCs with elevated major histocompatibility complex class II (MHCII) expression (MHCII^hi^) displayed molecular features of reduced cycling and apoptosis and was refractory to 5-fluorouracil–induced myeloablation. Conversely, MHCII^lo^ HSCs displayed increased megakaryocytic potential and were preferentially expanded in CALR mutant mice with thrombocytosis. Similar to mice, high MHCII expression is a feature of human HSCs residing in a deeper quiescent state. Our results therefore position STAT1 at the interface of stem cell heterogeneity and the interplay between stem cells and the adaptive immune system, areas of broad interest in the wider stem cell field.

## Introduction

Lifelong production of all mature blood and immune cells is sustained by a rare population of bone marrow hematopoietic stem cells (HSCs) that differentiate to produce a hierarchy of progenitors and mature cells.^1^ In steady state, although daily hematopoiesis is mainly maintained by actively cycling progenitors downstream of HSCs,^2–4^ the HSCs themselves are predominantly quiescent and thus largely protected from genotoxic insults.^5–8^ However, in response to acute stress such as blood loss, infection, or cytotoxic insults, HSCs can rapidly respond by temporarily exiting quiescence and activating cell division to ensure efficient replenishment of blood and immune cells.^9,10^ The behavior and integrity of HSCs are tightly regulated by intrinsic and extrinsic factors including the bone marrow environment, whereas dysregulation leads to hematopoietic failure and/or hematologic malignancies.^11–13^

Inflammation is a key regulator of HSC fate, and a growing body of studies has documented roles for inflammatory signals in modulating HSC fate and long-term functionality.^11,14–16^ Interferons (IFNs) are a family of inflammatory cytokines long considered to be antiproliferative^17,18^; indeed, IFN-α has been used as a therapy for cancer, particularly for some hematologic malignancies.^19–21^ However, activation of IFN-α signaling in HSCs was found to induce G_0_ exit and entry into active cell cycling,^10^ whereas HSCs lacking Irf2, a transcriptional suppressor of type I IFN signaling, showed enhanced cycling.^22^ Both studies reported that the activation of type I IFN signaling in HSCs led to impaired repopulation in transplantation assays.^10,22^ IFN-γ was also shown to activate HSC proliferation in vivo in response to bacterial infection.^23^ Interestingly, IFN-α-driven HSC proliferation was shown to be transient, and upon chronic exposure, HSCs return to quiescence, thus protecting them from exhaustion.^24^ In addition, IFNs trigger differentiation responses: IFN-α activates a posttranscriptional megakaryocytic program in a subset of HSC-like cells expressing high levels of the megakaryocytic marker CD41,^25^ whereas IFN-γ induces myeloid differentiation in a subset of HSCs expressing IFN-γ receptor.^26^

STAT1 is a pivotal downstream mediator of IFN signaling in the context of microbial infection or recognition of tumor cells.^27,28^ STAT1-deficient mice are born at normal frequencies with no gross developmental defects.^29,30^ However, STAT1 deficiency in human patients is associated with predisposition to mycobacterial and viral diseases,^31^ STAT1KO mice die of infection upon bacterial or viral challenge, and STAT1KO bone marrow macrophages and spleen cells fail to respond to IFN.^29,30^ STAT1KO mice produce normal numbers of B lymphocytes, monocytes, and granulocytes in fetal liver or neonatal thymus^29^ but have abnormal development of regulatory T cells (Treg) and natural killer cells.^32–34^ STAT1 was shown to be required for both IFN-α- and IFN-γ–induced HSC exit from dormancy,^10,23^ whereas loss of STAT1 had subtle effects on baseline HSC numbers and engraftment in primary transplants.^26^ However, the underlying mechanisms remain unclear, and particularly little is known about the role of STAT1 in regulating hematopoietic stem and progenitor cells (HSPCs) under steady-state conditions.

## Methods

### Mice

The wild-type C57BL/6 (CD45.2), C57BL/6.SJL (CD45.1), and CD45.1/CD45.2 F1 mice in this study were used at 10 to 24 weeks of age. STAT1^−/−^ mice^29^ were kindly gifted from Thomas Decker and were analyzed between the ages of 10 and 52 weeks. *Vwf*-eGFP mice^35^ were kindly gifted from Claus Nerlov and Sten Eirik Jacobsen. CALR^del^ knock-in mice were generated in the Green laboratory.^36^ All mice were on a C57BL/6 background and kept in specific pathogen-free conditions, and all procedures were performed according to UK Home Office regulations.

### 5-Fluorouracil (5-FU) treatment

5-FU (Sigma) was prepared in phosphate-buffered saline and administered intraperitoneally to STAT1KO or wild-type (WT) mice (150 mg/kg). Peripheral blood was collected via tail vein into EDTA-coated tubes for full blood counts at 0, 4, 8, 9, 10, 11, and 14 days after 5-FU administration.

### Smart-seq2 and HSPC 10X Genomics single-cell RNA sequencing (scRNA-seq)analysis

Single ESLAM (EPCR^+^CD45^+^CD150^+^CD48^−^) HSCs were fluorescence-activated cell sorting (FACS) sorted from bone marrow mononuclear cells (BMMNCs) and processed using Smart-seq2.^37^ Lineage^-^c-Kit^+^ (LK) cells were sorted from BMMNCs and processed according to the manufacturer's protocol for 10x Chromium (10X Genomics, Pleasanton, CA).

### Statistics

The statistical differences were assessed using a 2-tailed, unpaired Student *t* test unless otherwise indicated.

## Results

### Loss of STAT1 alters the steady-state landscape of HSPCs

**Figure 1:**
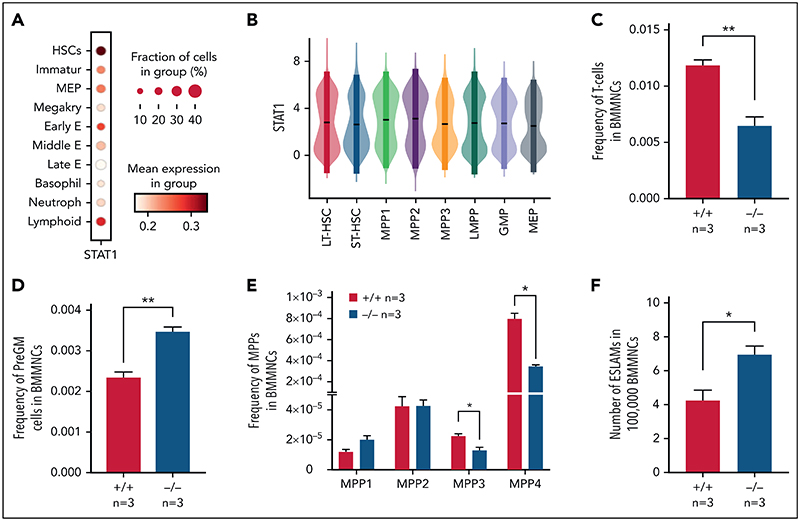
Loss of STAT1 affects the stem and progenitor compartment in steady-state hematopoiesis. (A) Dot plot showing normalized STAT1 expression in cell types across the Dahlin landscape. The size of each dot indicates the proportion of cells with normalized expression level >0, and the color intensity shows the levels of STAT1 expression. (B) Violin plots showing normalized STAT1 expression in immature cell types in Nestorowa's scRNA-seq dataset. Mean ± standard deviation indicated in overlaid box. (C) The frequency of T cells was reduced in STAT1-deficient bone marrow. (D) The frequency of pre-granulocyte-macrophage progenitors (PreGM) was increased in STAT1-deficient bone marrow. Flow cytometry was performed, and PreGM progenitors were defined as Lin^-^Sca1^-^cKit^+^CD41^-^CD16/32^-^CD105^-^CD150^-^. (E) The frequencies of MPP3 and MPP4 were reduced in STAT1-deficient bone marrow. Flow cytometry was performed, and multipotent progenitor MPPs were defined as the following: MPP1 (Flk2^-^CD150^+^CD48^-^LSK), MPP2 (Flk2^-^CD150^+^CD48^+^LSK), MPP3 (Flk2^-^CD150^-^CD48^+^LSK), and MPP4 (Flk2^+^CD150^-^CD48^+^LSK). (F) The frequency of ESLAM HSCs was increased in STAT1-deficient mice. Bone marrow ESLAM HSCs were defined as CD45+CD150+CD48^-^EPCR+ cells. Data are shown as mean ± standard error; asterisks indicate significant differences by Student *t* test (*P < .05; **P < .01).

To investigate the role of STAT1 in the HSPC compartment, we first analyzed its expression across immature and mature HSPC populations in previously published scRNA-seq datasets. We observed the highest expression of *Stat1* and prototypical pSTAT1 target genes in HSCs within the LK population,^38^ whereas relatively lower levels were seen in neutrophil, basophil, MK, and mid/late erythroid progenitors (Figure 1A; supplemental Figure 1A, available on the *Blood* Web site). Using scRNA-seq data of phenotypically defined HSPC populations,^37^ we confirmed that both LT-HSCs and the most immature cell populations express high levels of Stat1 (Figure 1B).

STAT1-deficient mice showed normal peripheral blood counts, spleen weight, and bone marrow cellularity (supplemental Figure 1B-D). Compared with WT controls, the bone marrow of STAT1-deficient mice contained similar frequencies of erythroblasts (CD71^+^Ter119^+^), megakaryocytes (CD41^+^CD42d^+^), myeloid (Ly6g^+^CD11b^+^) and B220^+^ B cells (supplemental Figure 1E-G), but the frequency of T cells (CD3e^+^) was reduced (Figure 1C). There was an increased frequency of myeloid progenitors (PreGM, Lin^-^Sca1^-^cKit^+^CD41^-^CD16/32^-^CD105^-^ CD150^-^) but no change in the other progenitors analyzed (Figure 1D; supplemental Figure 1H). Within the immature cell populations, the frequencies of myeloid-primed MPP3 and lymphoid-primed MPP4 compartments were decreased (Figure 1E), whereas the frequency of ESLAM HSCs^39^ was increased in STAT1-deficient mice (Figure 1F). Taken together, these data indicate that loss of STAT1 affects the HSPC compartment in steady-state hematopoiesis.

Single-cell RNA profiling enables high-resolution analysis of heterogeneous stem/progenitor populations.^1^ We therefore performed droplet-based 10X Genomics scRNA-seq to analyze how loss of STAT1 affects the transcriptomic landscape in bone marrow HSPCs. LK cells, containing the majority of hematopoietic progenitor cell populations, were sorted and sequenced from a pair of STAT1KO and control mice. Cells were projected onto a previously published LK dataset of 44 82 cells^38^ (supplemental Figure 2A). Cells from both STAT1KO and WT control mice were found in all major blood lineages. However, in STAT1-deficient mice, cells within the immature 2 cluster were relatively increased, whereas those in the stem/MPP, immature 1 and 3, and other lineage-restricted progenitor clusters were all decreased (supplemental Figure 2B-C).

These results therefore demonstrate that loss of STAT1 causes widespread alterations across the HSPC compartment and suggests that although immunophenotypically defined HSC numbers were increased in STAT1-deficient bone marrow, the size of the functional HSC pool may be reduced.

### HSCs from STAT1-deficient mice are functionally impaired in competitive transplantation assays

**Figure 2:**
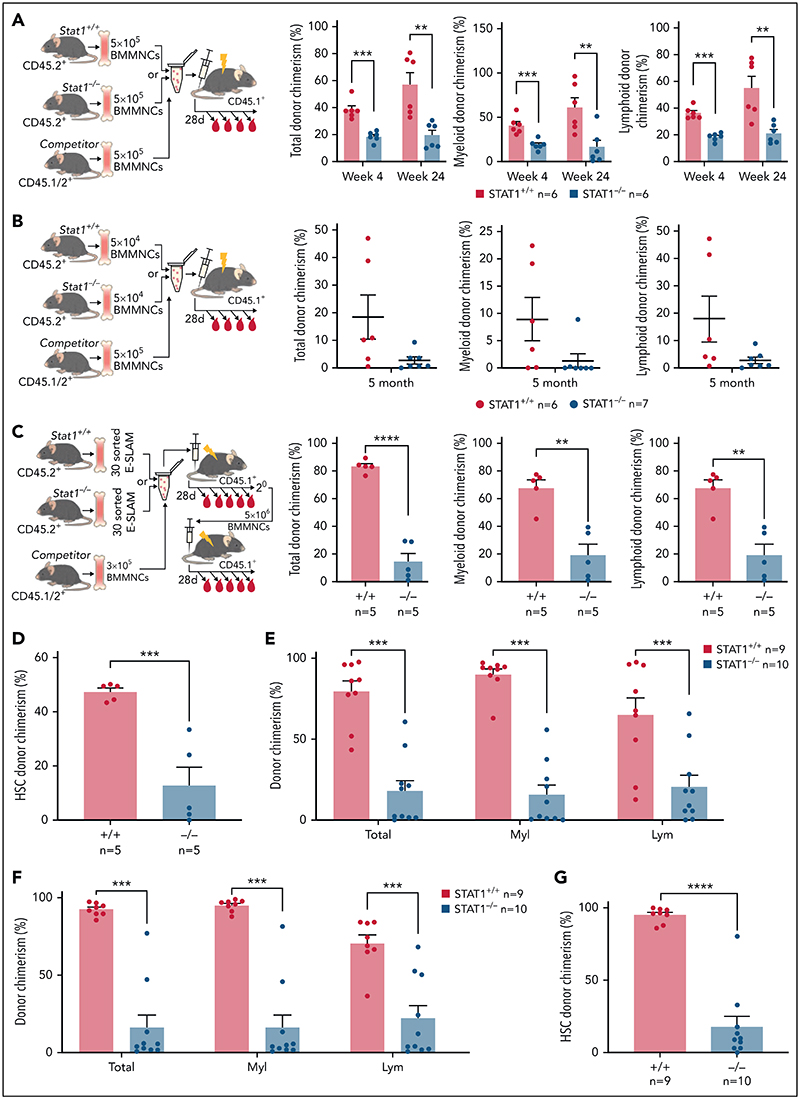
HSCs from STAT1KO mice show functional defects in competitive transplants. (A) STATI-deficient bone marrow (BM) cells exhibited reduced repopulating capacity in competitive transplant recipients. 5 × 10^5^ BM cells (CD45.2^+^) from STAT1KO or WT control mice were mixed with an equal number of competitor cells (CD45.1^+^/45.2^+^) and transplanted into lethally irradiated CD45.1 recipient mice. Donor repopulation was assessed using flow cytometry of nucleated peripheral blood with antibodies for CD45.1 and CD45.2 to distinguish donor origin: Ly6g and Mac1 for myeloid and B220 and CD3e for lymphoid cells. Bar graphs show the competitive repopulating ability of donor cells presented as the percentage of repopulated cells derived from test donor cells among the total number of donor-derived cells (y = test/[test + competitor]). (B) Bone marrow cells from STAT1KO mice contained a lower number of functional HSCs as shown by chimerism at 5 months posttransplantation. Competitive bone marrow transplantation was performed and analyzed as (A) using low-dose (5 × 10^4^) BM cells from WT or STAT1KO mice. At 5 months posttransplantation, 6 out 7 recipients receiving STAT1KO BM cells were found to have donor chimerisms <0.5% in myeloid lineage (5 with 0% and 1 with 0.2%), whereas only 2 recipients receiving STAT1^+/+^ BM had chimerisms <0.5% (1 with 0% and 1 with 0.1%). (C) ESLAM HSCsfrom STAT1KO mice displayed reduced repopulation capacity. 30 ESLAM HSCs FACS isolated from STAT1KO or WT control mice and mixed with 3 × 10^5^ CD45.1^+^/CD45.2^+^ competitor bone marrow cells were transplanted into lethally irradiated CD45.1 recipients. Repopulating capacity in bone marrow was analyzed as in (A). (D) Frequency of ESLAM HSCs derived from STAT1KO donor was reduced. At 6 months posttransplantation in (C), bone marrow cells from the recipient mice were assessed for donor-derived HSC chimerism using flow cytometry. ESLAM HSC was defined as CD45^+^CD150^+^CD48^-^EPCR^+^, and donor origin was distinguished using antibodies for CD45.1 and CD45.2. (E-F) ESLAM HSCs from STAT1KO mice displayed reduced repopulation capacity in blood (E) and bone marrow (F) in secondary transplant. 5 × 10^6^BMcells from the primary recipients in (C) were transplanted into secondary recipients (CD45.1^+^), and donor repopulation was assessed as in (A). (G) Frequency of ESLAM HSCs derived from STAT1KO donor was reduced in secondary transplant recipients at 5 months posttransplantation. Data are shown as mean ± standard error; asterisks indicate significant differences by Student *t* test (*P < .05; **P < .01; ***P < .001; ****P < .0001).

To investigate HSC function, we performed competitive transplantation assays. We first examined the repopulating capacity of bone marrow (5 × 10^5^ BMMNCs) from STAT1KO or WT control mice (CD45.2^+^) when transplanted into lethally irradiated recipients with an equal number of BMMNCs from C57B/L mice (CD45.1^+^/CD45.2^+^ F1). Recipient mice receiving STAT1KO BMMNCs showed lower levels of both myeloid and lymphoid chimerism (Figure 2A). When a lower dose of bone marrow (5 × 10^4^ BMMNCs) was transplanted, 6 out of 7 recipient mice receiving STAT1KO BMMNCs showed almost no repopulation, whereas multiple mice receiving WT cells showed donor chimerism >10% (Figure 2B). These data indicate that STAT1-deficient bone marrow contained lower numbers of functional HSCs.

We next performed competitive transplants using 30 FACS-isolated ESLAM HSCs from WT or STAT1KO mice. Recipients of STAT1KO HSCs showed reduced multilineage repopulation (Figure 2C) and a threefold reduction in donor-derived HSC chimerism (Figure 2D). Following secondary transplantation, we observed approximately fourfold lower multilineage repopulation and donor-derived HSC chimerism (Figure 2E-G). Collectively, these data demonstrate that loss of STAT1 impairs the ability of HSCs to undergo multilineage repopulation and self-renew.

### STAT1 is required to maintain protective transcriptional programs in homeostatic HSCs, including inhibition of cell cycling

**Figure 3:**
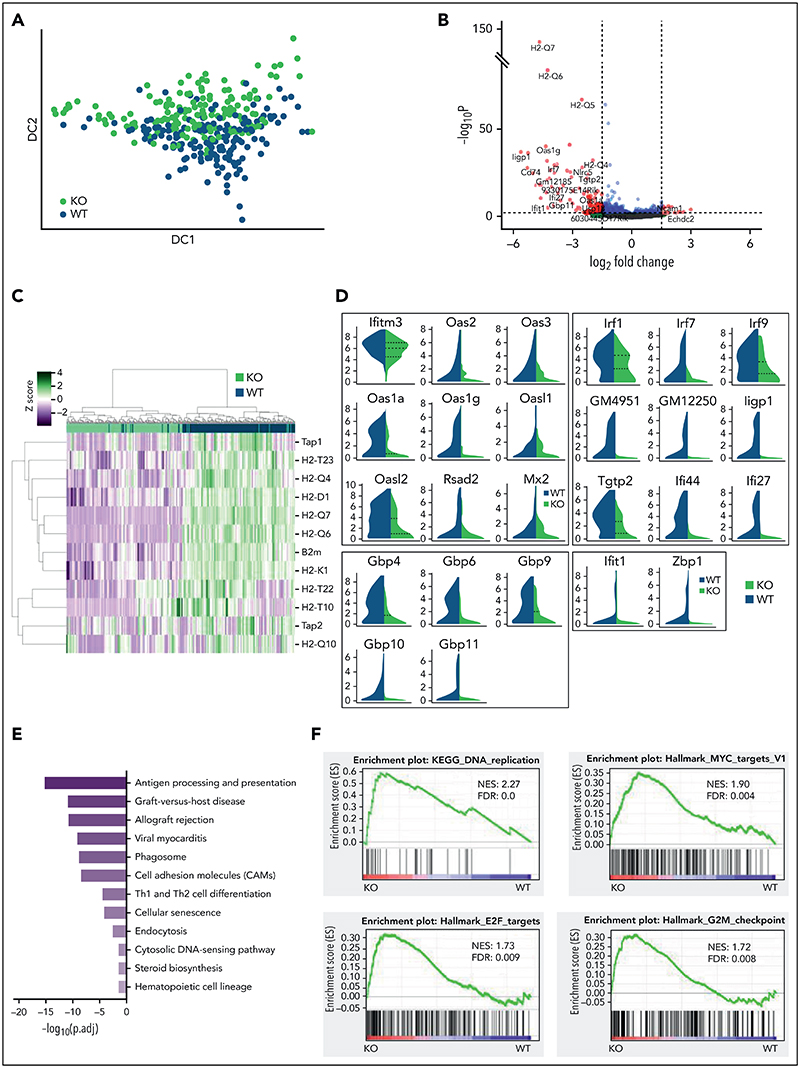
STAT1 is required to maintain protective transcriptional programs in homeostatic HSCs including inhibition of cell cycling. (A) Diffusion map showing a proportion of STAT1-deficient HSCs occupy space distinct from WT HSCs. WT ESLAM, dark blue dots; STAT1KO ESLAM HSCs, light green dots. (B) Volcano plot of differentially expressed genes (red dots) using DESEq2 and Benjamini-Hochberg corrected P values at a significance level of .01. (C) Heatmap showing MHCI gene expression and hierarchical clustering of ESLAM HSCs from STAT1KO or WT mice. (D) STAT1-deficient HSCs expressed reduced levels of genes involved in virus life cycle, viral sensing, and genes in pathways that activate transcription of IFN and IFN-stimulated genes. Violin plots showing normalized expression. (E) Pathway enrichment analysis showing downregulated Kyoto Encyclopedia of Genes and Genomes (KEGG) pathways in STAT1-deficient ESLAM HSCs. Statistical significance is indicated by −Log_10_(*P*.adj). (F) Gene set enrichment analysis (GSEA) plots showing significant enrichment of cell cycle related signatures in STAT1-deficient ESLAM HSCs. NES and FDR are indicated. FDR, false discovery rate; KO, knockout; NES, normalized enrichment score; *P*.adj., adjusted *P* value.

To interrogate the molecular programs controlled by STAT1 in steady-state HSCs, we sorted ESLAM HSCs from STAT1KO and WT mice and performed plate-based single-cell RNA sequencing. A total of 192 single HSC transcriptomes were generated for each genotype, of which 186 STAT1KO and 191 WT passed quality control (supplemental Figure 3A). These populations occupied distinct and overlapping spaces in diffusion maps (Figure 3A). Differential gene expression analysis identified 351 significantly downregulated genes and only 66 upregulated genes in STAT1KO HSCs (*P* < .05; supplemental Table 1). The magnitude of fold changes was generally much higher for downregulated genes than for the upregulated genes (Figure 3B). The most affected genes included a repertoire of molecules involved in antigen processing and presentation, including genes for the major histocompatibility complex (MHC) (Figures 3B-C). Markedly downregulated genes also included those involved in virus life cycle (*Ifitm3*, *Oas* family, *Mx2*, and *Dsad2*), IFN-stimulated genes, virus sensing genes (*Ifit1*, *Zbp1*), genes involved in the transcriptional response to IFN (*Irf1*, *Irf7*, and *Irf9*), and genes encoding AP-1 transcription factors (Figure 3D; supplemental Figure 3B).

We performed Gene Ontology (GO) pathway enrichment analyses using the lists of differentially expressed genes (cutoff of adjusted *P*< .05) and identified 23 GO terms that were significantly depleted in STAT1KO HSCs (cutoff q < 0.01; supplemental Table 2). These terms included antigen processing/presentation, response to IFNs, defense response to virus, and allograft rejection (Figure 3E; cholesterol biosynthetic process and secondary alcohol biosynthetic response, endoplasmic reticulum stress, and cell cycle arrest were also among those significantly downregulated terms (supplemental Table 2). In contrast, no pathways were significantly enriched in STAT1KO HSCs.

Consistent with GO analysis, GSEA of STAT1KO HSCs revealed depleted transcriptional signatures in response to IFNs, allograft rejection, inflammatory response, and cholesterol homeostasis (supplemental Figure 3C; supplemental Table 3). Conversely, signatures related to cell cycling were enriched in STAT1KO HSCs, including DNA replication, ribosome, Myc targets, E2F targets, and G2M checkpoint (Figure 3F). Conversely, genes related to cell cycle arrest were moderately reduced in STAT1-deficient HSCs (supplemental Figure 3D).

Together, these data demonstrate that STAT1-deficient HSCs at steady state have reduced expression of MHC molecules, IFN-stimulated genes, genes involved as defense against viral infection, and those involved in viral sensing/tumor immuno-surveillance. Our data also show that loss of STAT1 dysregulates several pathways that modulate stem cell behavior, including cholesterol biosynthesis,^40,41^ endoplasmic reticulum stress,^42^ and cell cycle.^6,7,43^

### STATI-deficient mice show delayed peripheral blood cell regeneration following myeloablation

**Figure 4:**
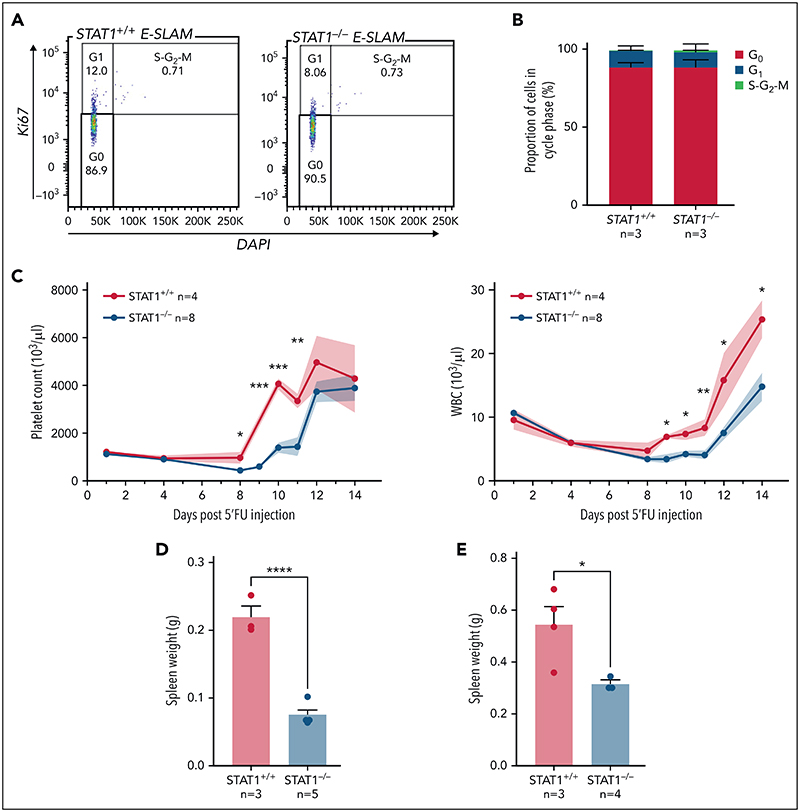
STAT1-deficient mice display delayed blood rebounds following 5'-FU-induced myeloablation. (A) Representative flow cytometry plots showing cell cycle analysis using intracellular staining of Ki-67/DAPI. G_0_ phase is defined as Ki-67^−^ and 2n DNA, G1 as Ki-67^+^ and 2n DNA, and S-G_2_-M as Ki-67^+^ and DNA > 2n. (B) Bar graphs showing comparable cycling status in ESLAM HSCs from STAT1KO and WT control mice. (C) STAT1-deficient mice showed delayed rebounds of platelets and white blood cells (WBC) following a single dose of 5-FU injection (150 mg/kg). (D-E) Bar graphs showing reduced spleen size in STAT1-deficient mice at days 12 and 15, respectively. Data are shown as mean ± standard error; asterisks indicate significant differences by Student *t* test (****P < .0001; ***P < .001; **P < .01; *P < .05).

The increased cell cycle signatures in STAT1-deficient HSCs raised the possibility that STAT1 inhibits cell cycle entry. This would be consistent with our observation that STAT1KO mice harbor increased numbers of immunophenotypic HSCs (Figure 1F) but fewer functional HSCs (Figure 2). To explore this possibility, we evaluated the cell cycle status of HSCs from STAT1-deficient mice under steady-state conditions. Flow cytometry using intracellular Ki-67/4,6-diamidino-2-phenylindole (DAPI) staining showed that the fraction of STAT1-deficient ESLAM HSCs in G_0_ was comparable to that from WT controls (Figure 4A-B). However, increased cycling of a subset of cells within a largely quiescent population may not be detectable by this approach.

We therefore employed 5-FU–induced myeloablation to activate dormant HSCs.^6,9,10,44,45^ Mice treated with a single dose of 5-FU were monitored for 14 days to establish the kinetics of WBC and platelet rebounds.^46,47^ Platelet and WBC rebounds began 8 days post–5-FU in WT mice, whereas rebounds in STAT1-deficient mice were significantly delayed (Figure 4C; supplemental Figure 4A). Although WT mice developed splenomegaly following 5-FU challenge as previously reported, STAT1-deficient mice had smaller-sized spleens at days 12 and 15 (Figure 4D-E) and showed increased proportions of lineage progenitors in bone marrow (supplemental Figure 4B). Despite the expansion of HSCs in steady-state STAT1-deficient mice, following 5-FU, STAT1-deficient and WT mice showed comparable numbers of HSCs to WT at days 12 and 15 (supplemental Figure 4C). Together, these observations are consistent with the notion that STAT1-deficient mice have increased numbers of cycling HSCs and fewer quiescent functional HSCs.

### STAT1 is essential for maintenance of MHCII^hi^ HSCs

**Figure 5:**
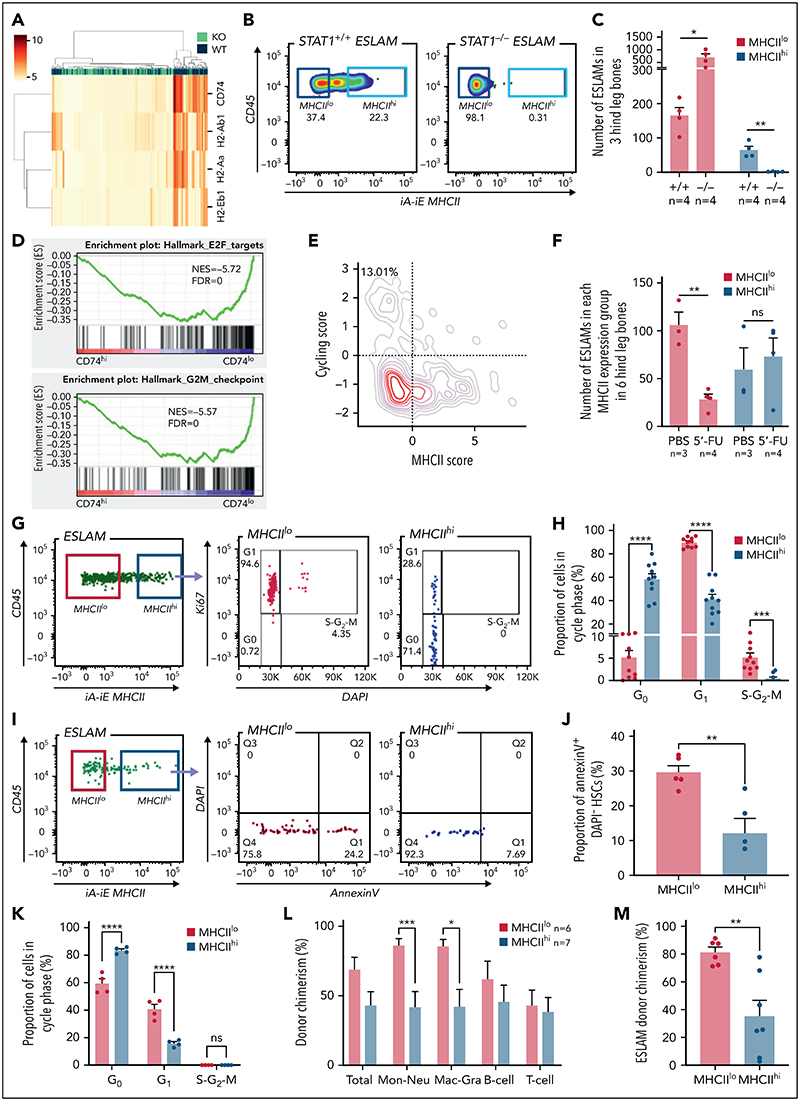
STAT1 maintains MHCII expression in a subset of HSCs (MHCII^hi^) that are refractory to myeloablation. (A) Heatmap showing MHCII gene expression and hierarchical clustering of ESLAM HSCsfrom STAT1KO and WT mice. (B) Representative flow cytometry plots showing MHCII expression on cell surface of HSCs, which was lost in STAT1-deficient ESLAM HSCs. (C) Bar graph showing the subsetof HSCswith high surface expression (MHCII^hi^) was completely lost in STAT1-deficient mice. (D) GSEA plots showing a depletion of cell cycle signatures in CD74^hi^ LT-HSCs. (E) LT-HSCs with low MHCII scores tended to display higher cycling scores. LT-HSCs from Nestorowa scRNA-seq dataset were analyzed. (F) Bar graph showing the subset of HSCs with low surface expression (MHCII^lo^) are preferentially depleted following a single dose of 5-FU treatment (150 mg/Kg). Flow cytometric analysis was performed on BMMNCs at 43 hours post-injection. (G) Representative flow cytometry plots showing cycling status for MHCII^hi^ andMHCII^lo^ HSCs following 5-FU treatment. (H) Bar graphs showing MHCII^hi^ HSCs display reduced cycling in response to 5-FU. (I) Representative flow cytometry plots showing apoptosis status for MHCII^hi^ and MHCII^lo^ HSCs following 5-FU treatment. (J) Bar graphs showing MHCII^hi^ HSCs displayed reduced apoptosis in response to 5-FU. (K) Bar graphs showing MHCII^hi^ HSCs display reduced cycling in response to polyinosinic–polycytidylic acid at 16 hours post-treatment. (L) Bar graphs showing donor chimerisms in peripheral blood at 16 weeks post-transplantation as analyzed in Figure 2. (M) Bar graphs showing reduced donor-derived ESLAM HSC chimerisms in recipient bone marrow at 16weeks post-transplantation. Data are shown as mean ± standard error; asterisks indicate significant differences by Student *t* test (****P < .0001; ***P <.001;**P <.01;*P < .05). FDR, false discovery rate; iA-iE, MHCII antibody; NES, normalized enrichment score; ns, not significant.

Although constitutive MHC class II (MHCII) expression is conventionally viewed as being restricted to professional antigen-presenting cells, our scRNA-seq analysis revealed that all the classical MHCII genes were expressed in a subset of homeostatic WT ESLAM HSCs, which was lost in STAT1-deficient HSCs (Figure 5A; supplemental Figure 5A). To investigate levels of MHCII expression within the HSPC compartment in more detail, we analyzed previously published scRNA-seq datasets.^37,38^ Within the LK population,^38^ there were higher levels of MHCII gene expression (except H2-Ab1) in HSC/MPPs and lymphoid progenitors compared with other progenitors (supplemental Figure 5B). Within the more immature cell populations,^37^ MHCII expression was highest in LT-HSCs (supplemental Figure 5C). Flow cytometric analysis demonstrated that cell surface MHCII proteins were readily detected on a subset of WT ESLAM HSCs (~20%) and that this subset (MHCII^hi^) was completely lost in STAT1-deficient mice (Figure 5B-C). It is worth noting that nearly all of the expanded ESLAM HSCs seen in STAT1-deficient mice belonged to the MHCII^lo^ subset (Figure 5C).

STAT1 loss not only depletes the MHCII^hi^ HSC subset but also causes transcriptional changes within the remaining MHCII^lo^ cells. If MHCII^hi^ HSCs are excluded, comparison of the remaining STAT1KO and WT HSCs showed that the pathways downregulated by loss of STAT1 remained largely unchanged (supplemental Figure 5D), whereas the MHCII genes themselves were no longer detected as differentially regulated (supplemental Table 4).

CIITA is a key regulator of MHCII genes and is a transcriptional target of STAT1. However, STAT1 loss did not result in downregulation of the already low levels of *Ciita* in HSCs (supplemental Figure 5E-F). Moreover, in plasmodium infected mice,^48^ IFN-γ caused upregulation of CD74, MHCII genes, and *Stat1*, but *Ciita* was not upregulated (supplemental Figure 5G). These data indicate that MHCII gene expression may be regulated by STAT1 independently of altered *Ciita* expression. Consistent with this concept, lipopolysaccharide upregulates MHCII expression in dendritic cells, without affecting CIITA levels, through an AP-1 enhancer located upstream of the I-Aβ promoter.^49^ Interestingly, the genes encoding several AP-1 transcription factors were downregulated in STAT1KO HSCs (supplemental Figure 3B).

### MHCII^hi^ HSCs represent a quiescent subset that is less responsive to stress-induced proliferation

To understand if MHCII-expressing HSCs exhibit distinct molecular and cellular properties, and given that CD74 is essential in the assembly and trafficking of MHCII for antigen presentation,^50^ we compared CD74^hi^ and CD74^lo^ fractions within WT LT-HSCs from a published scRNA-seq dataset^37^ (supplemental Figure 6A). GSEA analysis revealed that CD74^hi^ HSCs were enriched for IFN response signatures (supplemental Figure 6B) and depleted for cell cycle signatures (Figure 5D). Consistent with this, HSCs with low MHCII scores tend to display higher cycling scores (Figure 5E), a finding confirmed by analysis of an independent HSC scRNA-seq dataset^51^ (supplemental Figure 6C). However, Ki-67/DAPI staining did not reveal significant differences in cell cycle status between MHCII^hi^ and MHCII^lo^ HSCs from WT mice at steady state (supplemental Figure 6D).

We therefore considered the possibility that a subset of HSCs with high levels of MHCII expression and downregulated cell cycle signatures may be protected from stress-induced proliferation. Mice were challenged with 1 dose of 5-FU and analyzed for the activities of MHCII^hi^ and MHCII^lo^ HSCs. Following 5-FU, although MHCII^lo^ HSCs were preferentially depleted, MHCII^hi^ HSCs were maintained (Figure 5F; supplemental Figure 6E), and Ki-67/DAPI staining (supplemental Figure 6F) showed that, whereas almost all MHCII^lo^ ESLAM HSCs were driven out of G_0_, nearly 60% of MHCII^hi^ ESLAM HSCs remained in G_0_ (Figures 5G-H). Moreover, CD74^hi^ LT-HSCs showed downregulated apoptosis pathways in GO analysis (supplemental Figure 6G; supplemental Table 5), and MHCII^hi^ ESLAM HSCs displayed significantly lower rates of apoptosis compared with MHCII^lo^ HSCs both at steady state (supplemental Figure 6H) and following 5-FU treatment (Figure 5I-J). Consistent with these data, polyinosinic–polycytidylic acid treatment resulted in significantly more MHCII^hi^ HSCs remaining quiescent (Figure 5K), and singlecell assays showed that MHCII^hi^ HSCs exhibited delays in cell cycle entry in in vitro culture (supplemental Figure 6I).

To understand if MHCII^hi^ HSCs display distinct functional output in vivo, we performed competitive transplants using equal numbers of FACS-isolated MHCII^hi^ and MHCII^lo^ ESLAM HSCs from steady-state WT mice. Both MHCII^hi^ and MHCII^lo^ subsets contained functional stem cells, capable of multilineage blood repopulation, but MHCII^hi^ HSCs gave rise to lower levels of myeloid repopulation (Figure 5L; supplemental Figure 6J). At 16 weeks posttransplantation, recipient bone marrow analysis showed that donor-derived HSC chimerism was lower in MHCII^hi^ HSCs recipients, although this was not significant in both cohorts (Figure 5M; supplemental Figure 6K).

Taken together, these results demonstrate that both MHCII^hi^ and MHCII^lo^ subsets contain functional stem cells and that MHCII^hi^ HSCs represent a more quiescent subset, less responsive to stress-induced proliferation and apoptosis and which displays reduced myeloid repopulation and self-renewal in primary recipients.

### MHCII^lo^ HSCs exhibit enhanced megakaryocytic differentiation and are preferentially expanded in mutant CALR mice with thrombocytosis

**Figure 6:**
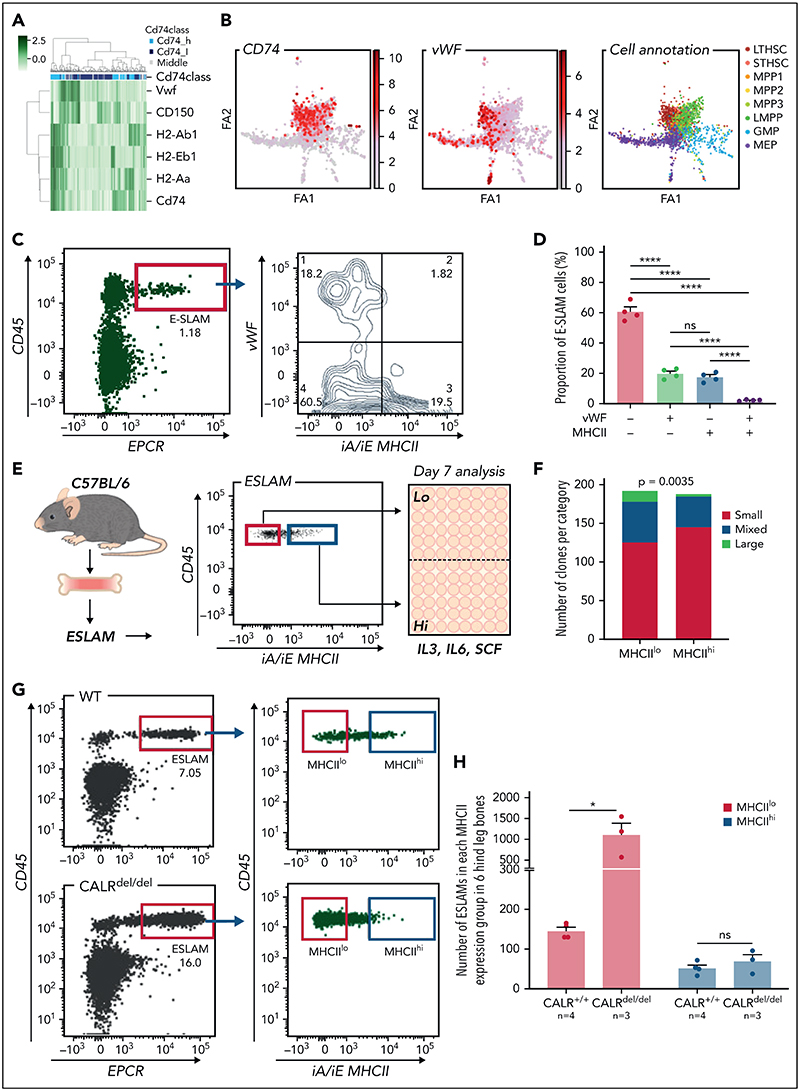
MHCII^lo^ HSCs exhibit enhanced megakaryocytic differentiation and are preferentially expanded in mutant CALR mice with thrombocythemia/myelofibrosis. (A) Heatmap showing Vwf-expressing HSCs cluster separately from HSCs with high levels of MHCII gene expression. LT-HSCs from Nestorowa scRNA-seq dataset were analyzed; a large proportion of LT-HSCs expressing high levels of Vwf is shown to cluster separately from HSCs with high-level expression of MHCII genes. (B) Expression of Cd74, Vwf genes plotted on the force-directed graph generated from HSPC cells in Nestorowa's scRNA-seq dataset. (C) Representative flow cytometry plots showing Vwf^+^ HSCs were within the MHCII^lo^ fraction. (D) Bar graphs showing the negative correlation between Vwf and MHCII cell surface expression within ESLAM HSCs. (E) Experimental scheme showing single-cell in vitro assays of ESLAM HSC differentiation. Single ESLAM HSCs gated with MHCII^hi^ or MHCII^lo^ were FACS sorted into 96-well plates and cultured in StemSpan medium with 10% fetal bovine serum (FBS), 250 ng/mL stem cell factor (SCF), 10 ng/mL IL-3, and 10 ng/mL IL-6, and at day 7, each individual cell-derived clone was scored and categorized using criteria as described in Prins et al.^54^ (F) Bar graphs showing a reduced number of clones derived from MHCII^hi^ ESLAM HSCs with presence of large cells at day 7. MHCII^hi^, n = 187 wells; MHCII^lo^, n = 193; Chi-squared test; *P* = .0035. (G) Representative flow cytometry plots showing increased frequency of MHCII^lo^ ESLAM HSCs in knock-in mice expressing homozygous mutant CALR (CALR^del/del^). (H) Bar graphs showing preferential expansion of MHCII^lo^ ESLAM HSCs in mutant CALR mice. Data are shown as mean ± standard error; asterisks indicate significant differences by Student *t* test (*P < .05; ****P < .0001). iA/iE, MHCII antibody; Large, colonies of any cell number (usually 1-30 cells), containing only very large flattened cells; Mixed, colonies of any cell number, containing small round cells and very large flattened cells; ns, not significant; Small, colonies of any cell number, containing cells that are uniformly round and small; STHSC, short term HSCs.

A heatmap displaying expression of MHCII genes together with CD150 and *Vwf* (both associated with specific lineage biases^35,52^) showed that HSCs expressing MHCII genes clustered separately from *Vwf* expressing HSCs (Figure 6A). Moreover, plotting the abundance of MHCII genes on a force-directed graph generated from the Nestorowa scRNA-seq dataset^37^ revealed distinct trajectories for MHCII and Vwf expression (Figure 6B). Analysis of HSCs from Vwf-GFP mice^35^ showed that the most Vwf-GFP^+^ ESLAM HSCs were MHCII^lo^ (Figures 6C-D). These data suggested that MHCII^hi^ HSCs may display reduced megakaryocytic differentiation.

Consistent with this idea, CD74^hi^ LT-HSCs showed downregulated megakaryocytic differentiation by GO analysis (supplemental Figure 7A), and flow cytometry analysis of HSCs revealed a negative correlation between expression of MHCII and expression of c-Kit, CD41, or CD150, which are markers expressed at higher levels in Mk-biased HSCs.^25,53^ (supplemental Figure 7B). Furthermore, clones derived from single MHCII^hi^ HSCs (cultured in conditions permissive for megakaryocyte differentiation as previously described^54^ [Figure 6E]) showed less megakaryocytic differentiation than those derived from MHCII^lo^° HSCs (Figure 6F).

Somatic mutations in CALR are found in ~40% of patients with essential thrombocythemia and primary myelofibrosis. Knock-in mice expressing mutant CALR (CALR^del/del^) develop marked thrombocytosis, increased megakaryopoiesis, and an expansion of immunophenotypically defined HSCs.^36^ We considered the possibility that an altered balance of MHCII^hi^ and MHCII^lo^ HSCs might contribute to the increased megakaryopoiesis seen in CALR^del/del^ mice. Analysis of our scRNA-seq dataset^54^ showed lower levels of MHCII expression in LT-HSCs from CALR^del/del^ mice (supplemental Figure 7C) together with an increased proportion of MHCII^lo^ LT-HSCs (supplemental Figure 7D). Flow cytometry demonstrated that MHCII^lo^ HSCs were indeed preferentially expanded in CALR^del/del^ mice compared with WT controls, whereas the number of MHCII^hi^ HSCs remained unchanged (Figure 6G-H; supplemental Figure 7E).

To assess the in vivo functional output of mutant MHCII^hi^ and MHCII^lo^ HSCs, 50 FACS-sorted MHCII^hi^ or MHCII^lo^ ESLAM HSCs from CALR^del/del^ mice were mixed with 2 × 10^5^ BMMNCs and transplanted. Sixteen weeks posttransplantation, elevated platelet counts were seen in 3 out of 5 recipients of MHCII^hi^ HSCs and in 3 out of 5 recipients of MHCII^lo^ HSCs. Interestingly, recipients of MHCII^lo^ HSCs displayed a trend of higher platelet counts despite relatively lower total CD45.2^+^ chimerism (supplemental Figure 7F-H), suggesting both MHCII subpopulations are capable of driving the disease. Transplantation of either MHCII^hi^ or MHCII^lo^ donor HSCs from CALR^del/del^ mice gave rise to both subpopulations in recipient bone marrow (supplemental Figure 7I), indicating that both subsets were capable of inter-converting, although MHCII^hi^ donor HSCs were more likely to do so.

Together, these data demonstrate that the MHCII^hi^ HSC subset has a reduced potential to undergo megakaryocytic differentiation and that mutant CALR drives the preferential expansion of MHCII^lo^ HSCs that display increased megakaryocytic potential.

### MHCII high expression marks subset of HSCs with distinct functionality in human

**Figure 7:**
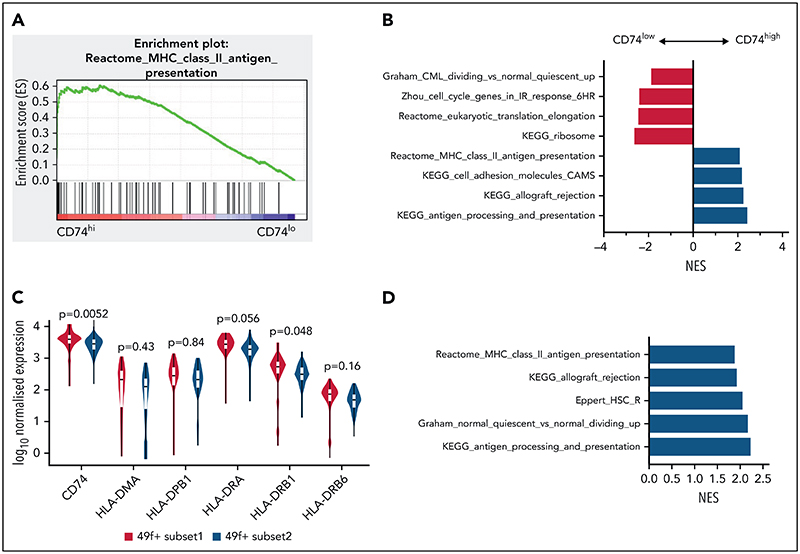
MHCII high expression marks a subset of HSCs in a deeper quiescent state in human. (A) Enrichment plot of Reactome: MHC Class II Antigen Presentation from GSEA analysis of human CB LT-HSCs comparing the top 30% of CD74 expression (CD74^hi^) and bottom 30% of CD74 expression (CD74^lo^). (B) Selected biological pathways (c2 curated pathways; FDR < 0.05) from preranked GSEA of human CB LT-HSCs with top 30% CD74 expression (CD74^hi^) compared with bottom 30% CD74 expression (CD74^lo^) (50 cells) from Belluschi et al.^55^ (C) Normalized expression of key MHCII regulators; FDR for differential expression between 49f^+^ Subset1 and 49f^+^ Subset2 as determined by DESeq2 shown. (D) Selected biological pathways (c2 curated pathways; FDR < 0.05) enriched in preranked GSEA analysis of 49f^+^Subset1 (CD34^lo^/C9A^hi^) and 49f^+^Subset2 (CD34^hi^/C9A^lo^) from Belluschi et al.^55^ FDR, false discovery rate; NES, normalized enrichment score.

To explore if differing levels of MHCII expression identify functionally distinct HSCs in humans, we reanalyzed a scRNA-seq dataset of human HSCs,^55^ where single HSCs were sorted on the most stringent CD49f^+^ CD90^+^ phenotype.^56^ Cells were classified by CD74 messenger RNA expression, where the top and bottom 30% were referred to respectively as CD74^hi^ and CD74^lo^ LT-HSCs. Gene sets related to MHCII presentation were significantly enriched in CD74^hi^ LT-HSCs compared with CD74^lo^ LT-HSCs (Figure 7A). Consistent with our mouse data, gene sets related to cell cycle were significantly depleted in CD74^hi^ LT-HSCs (Figure 7B).

We also found that gene sets related to MHCII regulation and expression of key regulators of MHCII antigen processing and presentation were significantly higher in LT-HSCs with high cell surface CLEC9A expression and low CD34 expression CLE-C9A^hi^CD34^lo^, noted as subset 1 (Figure 7C-D). Subset 1 LT-HSCs were functionally demonstrated by Belluschi et al^55^ to contain long-term repopulating multipotent HSCs with slow quiescence exit kinetics compared with subset 2 LT-HSCs (CLEC9A^lo^CD34^hi^), a subset restricted to myelo-lymphoid differentiation with infrequent but durable repopulation capacity.

Taken together, these results show that differing levels of CD74 and MHCII expression are associated with functionally distinct human HSCs. Consistent with our mouse data, MHCII high-expressing human HSCs displayed deeper quiescence.

## Discussion

STAT1 is well recognized to be essential for IFN-mediated activation of HSCs. Here, we show that STAT1 also regulates homeostatic HSPCs and is critical for HSC self-renewal and maintenance of transcriptional programs that protect HSC integrity. In addition, we report previously unrecognized HSC subsets with differing MHCII expression: STAT1-dependent MHCII^hi^ HSCs, which are less responsive to stress-induced proliferation, and MHCII^lo^ HSCs, which exhibit enhanced megakaryocytic differentiation potential and are preferentially expanded in a mutant CALR knock-in mouse model. Similar to mice, high MHCII expression is a feature of human HSCs residing in a deeper quiescent state.

STAT1-deficient mice harbored increased numbers of immunophenotypic HSCs, which showed impaired lymphoid and myeloid repopulation and self-renewal in serial competitive transplants. Increased proliferation of HSCs has previously been reported to accompany functional exhaustion.^6,7,43,57^ Our results indicate that homeostatic STAT1-deficient HSCs are transcriptionally primed for cell division, observed through the enrichment of cell cycle signatures. Furthermore, STAT1-deficient mice displayed delayed WBC and platelet rebounds following 5-FU, although concomitant effects on progenitors may also contribute to this delayed rebound. These results are consistent with a recent study, which reported that HSCs in STAT1KO mice were expanded but displayed reduced function after transplantation or 5-FU.^58^ Our scRNA-seq analysis showed that STAT1 loss altered several pathways that modulate HSC function, including cholesterol biosynthesis,^40,41^ endoplasmic reticulum stress,^42^ and cell cycle.^6,7,43^ STAT1 is known to be a key component of signaling pathways triggered by multiple cytokines including IFNs, and it is possible that interruption of autocrine positive feedback loops^59^ may contribute to the intrinsic functional defects of STAT1-deficient HSCs. We also considered the possibility that alterations in the cellular environment might contribute to the altered HSC function. However, in our primary and secondary recipients of STAT1KO HSCs, 80% to 90% of the bone marrow hematopoietic cells were WT, and in secondary recipients, there were no differences in the proportion of myeloid (Ly6G^+^ and CD11b^+^) and lymphoid (B220^+^ and CD3e^+^) cells (data not shown), suggesting that changes in the cellular environment are highly unlikely to account for the observed HSC functional defects.

Previous seminal studies have revealed that HSCs display functional heterogeneity with regards to self-renewal and lineage bias,^35,60–64^ although the underlying mechanisms remain largely unknown. Our results demonstrate the existence of functional HSC heterogeneity associated with MHCII expression and show that MHCII^hi^ HSCs were absent in STAT1 knockout mice. Our data do not exclude the possibility that MHCII^hi^ HSCs are lost due to enhanced differentiation from MHCII^hi^ into MHCII^lo^ HSCs. MHCII^hi^ HSCs displayed molecular features of reduced cycling and apoptosis and were resistant to 5-FU–induced proliferation. When the functional output of MHCII^hi^ HSCs were tested in transplants, MHCII^hi^ HSCs tended to display lower levels of myeloid repopulation and HSC chimerism, suggesting these cells were less active in repopulating and self-renewing. However, our data do not exclude the possibility that MHCII^hi^ HSCs display a better self-renewal potential over time upon further sequential transplantation.

A recent elegant study combining lineage tracing with single-cell transcriptomics^65^ demonstrated that, following 5-FU challenge, a fraction of HSCs did not produce progeny, termed ″childless″ HSCs. Examination of their transcriptomic data shows that MHCII genes are highly enriched in childless HSCs (see Figure 6 of Bowling et al^65^), which supports our findings that MHCII^hi^ HSCs were less responsive to 5-FU–induced proliferation. Another study from the same group reported that a subset of donor-derived HSCs that displayed low lineage output after transplantation were high in CD74; however, these same HSCs displayed a bias toward the Mk lineage.^66^ These findings contrast with our observation that MHCII^hi^ HSCs have reduced megakaryocytic bias and suggest that MHCII^hi^ HSCs may behave differently in the transplant setting. The specific absence of the MHCII^hi^ population in the STAT1 genetic knockout model afforded us the unique opportunity to interrogate its molecular and functional characteristics, an exploration that has hitherto proved difficult when studying the biological relevance of HSC heterogeneity.

scRNA-seq analysis of homeostatic HSCs revealed a negative correlation between the expression of MHCII genes and *Vwf*, which is known to be associated with megakaryocytic lineage bias.^35^ MHCII^hi^ HSCs also displayed reduced megakaryocytic differentiation compared with MHCII^lo^ HSCs, consistent with the clear separation of MHCII^hi^ HSCs from Vwf-expressing HSCs in flow cytometric analysis. These results led us to investigate whether MHCII^lo^ HSCs might contribute to the expansion of the megakaryocytic lineage found in the mutant CALR mouse model.^36^ These mice displayed a substantial expansion of MHCII^lo^ HSCs. Transplantation of purified MHCII^hi^ or MHCII^lo^ ESLAM HSCs from mutant CALR mice were both able to reconstitute the disease (elevated platelet counts) and gave rise to both subsets in recipient mice. Together, our results suggest a model in which mutant CALR drives a marked expansion of MHCII^lo^ HSCs but not MHCII^hi^ HSC and that there is also interconversion between MHCII^hi^ and MHCII^lo^ HSCs.

The function of MHCII on HSCs is unclear, but several lines of evidence raise the possibility that it may relate to a role for T cells in modulating HSC biology. Recent studies reported MHCII expression in Lgr5^+^ intestinal stem cells. Intestinal stem cell numbers were increased in MHCII-deficient or Treg-deficient mice.^67^ In addition, it was reported that Tregs in skin preferentially localize to hair follicle stem cells and promote hair follicle regeneration by augmenting hair follicle stem cell proliferation and differentiation.^68^ Of note, CD150^high^ bone marrow Tregs have been reported to support HSC quiescence as Treg depletion increased HSC numbers.^69^ Because impaired Treg development was reported in STAT1KO mice,^32^ it is tempting to speculate that loss of MHCII expression on HSCs and Treg dysfunction may both contribute to the HSC expansion and functional impairment that we have observed in STAT1-deficient mice. CIITA is the archetypal regulator of MHCII gene expression,^64^ and STAT1 mediates IFN-γ–induced MHCII expression by activating CIITA.^70^ Interestingly, we did not observe a significant downregulation of *Ciita* gene expression in STAT1-deficient HSCs from steady-state mice or upregulation of *Ciita* in LT-HSCs from mice infected with plasmodium. These findings suggest that STAT1 may regulate MHCII expression via mechanisms independent of *Ciita* induction. A complex picture therefore emerges whereby the direct activation of MHCII gene expression in HSCs by STAT1 may be accompanied by interactions with immune cells, which together contribute to the formation and/or maintenance of an HSC subpopulation with distinct molecular and functional characteristics.

## Supplementary Material

Supplemental methods and figures

Supplemental table 1

Supplemental table 2

Supplemental table 3

Supplemental table 4

Supplemental table 5

## Key Points

- STAT1 is essential for normal HSC function and maintenance of a MHCII^hi^ HSC subset that is less responsive to stress-induced proliferation.
- MHCII^hi^ and MHCII^lo^ subsets both contain functional HSCs, but MHCII^lo^ HSCs show increased Mk potential and are expanded in mutant CALR mice.
