# Supplementary material for: STAT1 is essential for HSC function and maintains MHCIIhi stem cells that resist myeloablation and neoplastic expansion
Source: Blood. 2022 Jul 16;140(14):1592–606. doi: 10.1182/blood.2021014009 (PMC7614316; doi:10.1182/blood.2021014009)
Supplement: Supplemental File 1 [file BLOOD_BLD-2021-014009-mmc1.pdf]

Supplemental Methods

Mice genotyping

Stat1 -/- mice (Durbin et al., 1996)

STAT1-NEO-R TAATGTTTCATAGTTGGATATCAT

STAT1-GENO-R CTGATCCAGGCAGGCGTT G

STAT1-GENO-F GAGATAATTCACAAAATCAGAGAG

Vwf-eGFP mice (Sanjuan-Pla et al., 2013)

Vwf2 Fw CCTCTCTGGACGGTGAGAAC

Vwf2 Rv AAGTCGTGCTGCTTCATGTG

CALRdel mice (Li et al., 2018)

CALR KI 19446F CCTACCTTCTCAGTGCATCAA

CALR KI 20223R ATCTGAACCTGCCTGGAAAA

Bone marrow cell harvest, Flow cytometric analysis

Tibia, femur and iliac crest bones were flushed or crushed in a mortar with FACS buffer (PBS/10%FBS/5mM EDTA), and were filtered through a 70-μm strainer to obtain single cell suspensions. Equal volume of Ammonium chloride solution (Stemcell Technologies) was mixed with cell suspension gently and incubated for 10 min at 4°C to lyse red blood cells. Cells were then spun at 360 xg for 5 min, and BMMNCs were washed in 40mL FACS buffer and

resuspended in an appropriate volume for antibody staining. The BMMNC were incubated with the appropriate dilution (2-5 mg/mL) of fluorescent antibody conjugates and 4',6-diamidino-2-phenylindole (DAPI) or 7AAD for dead cell exclusion, and analyzed on LSRFortessa flow cytometer (BD Biosciences, Franklin Lakes, NJ) equipped with FACSDiva Software. Data were analyzed using FlowJo (Tree Star, Ashland, USA).

The frequency of HSCs, progenitors and lineage cells in bone marrow was analyzed by flow cytometry: T cells, CD3e^{+}; B cells, B220^{+}; erythroblasts, CD71^{+}Ter119^{+}; myeloid cells, Ly6G^{+}Mac-1^{+}; MK, CD41^{+}CD42^{+}; prog, Lin^{-}Sca1^{-}cKit^{+}; megakaryocyte progenitors (MkP), Lin^{-}Sca1^{-}cKit^{+}CD150^{+}CD41^{+}; PreGM, Lin^{-}Sca1^{-}cKit^{+}CD41^{-}CD16/32^{-}CD105^{-}CD150^{-}; GMP, Lin^{-}Sca1^{-}cKit^{+}CD41^{-}CD16/32^{+}CD150^{-}; PreCFU-e, Lin^{-}Sca1^{-}cKit^{+}CD41^{-}CD16/32^{-}CD105^{+}CD150^{+}; CFU-e, Lin^{-}Sca1^{-}cKit^{+}CD41^{-}CD16/32^{-}CD105^{+}CD150^{-}; PreMegE, Lin^{-}Sca1^{-}cKit^{+}CD41^{-}CD16/32^{-}CD105^{-}CD150^{+} and PreGM, Lin^{-}Sca1^{-}cKit^{+}CD41^{-}CD16/32^{-}CD105^{-}CD150^{-}. Multipotent progenitor MPPs were defined as the following: MPP1 (Flk2^{-}CD150^{+}CD48^{-}LSK), MPP2 (Flk2^{-}CD150^{+}CD48^{+}LSK), MPP3 (Flk2^{-}CD150^{-}CD48^{+}LSK) and MPP4 (Flk2^{+}CD150^{-}CD48^{+}LSK). LTHSC was defined as Flk2^{-}, CD34^{-} Lin^{-}Sca1^{+}cKit^{+}CD150^{+}CD48^{-} and ESLAM HSCs as CD45^{+}EPCR^{+}CD150^{+}CD48^{-}.

FACS Isolation of E-SLAM HSCs for transplants

Single cell suspensions of BMMNCs were first lineage depleted using EasySep™ Mouse Hematopoietic Progenitor Cell Isolation Kit (STEMCELL Technologies). Cells were then stained with EPCR PE (STEMCELL Technologies), CD150 PE/Cy7 (BioLegend), CD45 FITC and CD48 APC (Biolegend). E-SLAM HSCs defined as $CD45^{+}EPCR^{+}CD48^{-}CD150^{+}$ were isolated using a Becton Dickinson Influx sorter (BD Biosciences).

Competitive transplantation assays

C57BL/6 (CD45.1+) and $CD45.1^{+}$ W41 ($cKit^{W41/W41}$) recipients were irradiated with 2 x 550 and 400 cGy respectively. For competitive repopulation assays, bone marrow cells (5 x 10^5 or lower dose 5 × 10^4) with 0.5 x 10^6 nucleated competitor BM cells obtained from CD45.1/CD45.2 F1 mice were injected into recipient mice. To evaluate qualitative differences of HSCs from WT and mutant mice or different subset of HSCs, equal number of FACS isolated ESLAM HSCs were mixed with 0.3 x 10^6 nucleated competitor BM cells obtained from CD45.1/CD45.2 F1 mice before being injected into $CD45.1^{+}$ recipient mice. At 16 weeks or longer post transplantation, bone marrow cells from the recipient mice were assessed for donor-derived HSC chimerism using flow cytometry by staining bone marrow cells with ESLAM markers as above as well as CD45.1 and CD45.2.

Secondary transplantation was then performed using 5 x 10^6 nucleated BM cells from primary recipients of ESLAM HSCs. In all cases, peripheral blood was obtained and analyzed by flow cytometry for donor contribution to myeloid (Ly6g and Mac1) and lymphoid (B220 and CD3e) lineages together with antibodies for CD45.1 and CD45.2 to distinguish the donor origin of repopulated cells.

Flow cytometric analysis and FACS isolation of MHCII^{hi} and MHCII^{lo} ESLAM HSCs

Single cell suspensions of BMMNCs were first lineage depleted using EasySep™ Mouse Hematopoietic Progenitor Cell Isolation Kit (STEMCELL Technologies). Then the cells were stained with ESLAM HSC markers as above and I-A/I-E APC antibody (Biolegend). Gating for MHCII^{hi} ESLAM HSCs were determined using STAT1KO ESLAM HSCs as a negative control due to lack of MHCII expression in STAT1-deficient HSCs.

MHCII^{hi} and MHCII^{lo} ESLAM HSC single cell *in vitro* differentiation assays

Single ESLAM HSCs were cultured and the derived clones were classified as previously described with minor modifications (Prins et al 2020). Briefly, single MHCII^{hi} and MHCII^{lo} ESLAM HSCs were FACS sorted into round-bottom 96-well plates (Corning, Corning, USA) preloaded with 50µl StemSpan SFEM (serum-free expansion medium) (STEMCELL Technologies). A further 50 µL of

medium was subsequently added to each well to a final concentration of 10% FBS (STEMCELL Technologies), 1% penicillin/streptomycin (Sigma-Aldrich), 1% l-glutamine (Sigma-Aldrich), stem cell factor (SCF; 250 ng/ml), IL-3 (10 ng/ml), IL-6 (10 ng/ml; STEMCELL Technologies) and 0.1 mM β-mercaptoethanol. At day 7 of culture, single cell–derived clones were visually inspected. Wells with surviving cells were classified into one of three categories: (i) wells containing only one or more enlarged cells, which were to become megakaryocytes as characterized (Prins et al., 2020) in this culture system; (ii) mixed expansion, with both small and enlarged cells; and (iii) expansion with only small cells. The experimenter was blinded to the identity of the cells sorted into the well.

Cell cycle analysis of steady state ESLAM HSCs

Bone marrow cells from untreated STAT1KO and WT control mice were lineage depleted using EasySep™ Mouse Hematopoietic Progenitor Cell Isolation Kit. The cells were then stained for 45 minutes with CD45 BV785 (Biolegend), EPCR PE (STEMCELL Technologies), CD150 PE/Cy7 (BioLegend), CD48 BV605 and Zombi Nir to eliminate dead cells. The stained cells were washed with FACS buffer (PBS/2% FBS), then fixed and permeabilized using Cytofix/Cytoperm™ Fixation/Permeablization kit (BD Biosciences). The cells were then stained with Ki-67 FITC (Biolegend) on ice for 1 hr and then with DAPI (Thermo Fisher) for 1 hour at RT. Flow cytometric analysis was carried out at a low flow rate using LSRFortessa flow cytometer (BD).

Cell cycle analysis of MHCII^{hi} and MHCII^{lo} ESLAM HSC following 5-FU or poly-IC treatment

5-FU (150mg/Kg) or poly-IC (10mg/Kg) was administered intraperitoneally (i.p.) to mice, at 70 hours post injection post 5-FU or 16 hours post poly-IC, bone marrow cells were harvested and stained with for 45 minutes with CD45 BV785 (Biolegend), EPCR PE (STEMCELL Technologies), CD150 PE/Cy7 (BioLegend), CD48 BV605, I-A/I-E antibodies and Zombi Nir to eliminate dead cells. The stained cells were washed with FACS buffer (PBS/2% FBS), then fixed and permeabilized using Cytofix/Cytoperm™ Fixation/Permeablization kit (BD Biosciences). The cells were then stained with Ki-67 FITC (Biolegend) on ice for 1 hour and then with DAPI (Thermo Fisher) for 1 hour at RT. Flow cytometric analysis was carried out at a low flow rate using LSRFortessa flow cytometer (BD).

Apoptosis analysis of ESLAM HSCs

Bone marrow cells from either steady state or 5-FU treated mice were mixed with equal volume of ammonium chloride (StemCell Technologies) and incubated on ice for 3 minutes to lyse red blood cells. BMMNCs were then stained with CD45 BV785 (Biolegend), EPCR PE (STEMCELL Technologies), CD150 PE/Cy7 (BioLegend), CD48 BV605, I-A/I-E APC (Biolegend) for 45 minutes. The stained cells were washed with FACS buffer (PBS/2% FBS), then

stained with 5ul Annexin V FITC (Biolegend) in 100ul PBS/2% FBC/5mMEDTA at room temperature in dark for 15 minutes. DAPI was then added to the samples (Thermo Fisher). Flow cytometric analysis was carried out using LSRFortessa flow cytometer (BD).

Analysis of division kinetics in Single HSC *in vitro* Cultures

E-SLAM HSCs were sorted into round-bottom 96-well plates, preloaded with 50 μL serum-free Stemspan medium (STEMCELL Technologies). A further 50 μL of medium was subsequently added to each well to a final concentration of 10% FCS, 1% Pen/Strep, 1% L-Glut, 250 ng/mL SCF (STEMCELL Technologies), 20 ng/mL IL-3 and IL-6 (Peprotech) and 0.1 mM β-mercaptoethanol. For cell division kinetics, the number of cells in each well was counted manually every day for up to 5 days in culture.

STAT1KO and WT ESLAM cells Smart-seq2 data analysis

Bone marrow cells were harvested from STAT1KO and WT control mice and single ESLAM HSCs were FACS sorted as described above and processed as described for Smart-seq2.

<u>Preprocessing</u>

The reads resulting from the SmartSeq2 experiments were mapped against Ensembl genes (release 81) (Zerbino et al., 2018) using GSNAP (version

2015-09-29) (Wu and Nacu, 2010) and quantified using HTSeq (version 0.6.0) (Anders et al., 2015). Filtering Quality Control (QC) steps were applied where nuclear genes had to be at least 20% of the mapping reads and cells with less than 50,000 reads mapping to them were rejected. In addition, the maximum allowed fraction of cells mapping to mitochondrial genes was set at 20%. The levels of technical variance were estimated using the ERCC spike-ins as described by Brennecke et al. (2013) with highly variable genes (HVGs) being defined as having the squared coefficient of variation exceeding technical noise. The resulting dataset was also transformed by applying the remove batch effect method implemented within the R limma package (Ritchie et al., 2015). The raw sequencing reads and gene count tables were deposited at the NCBI GEO (accession number: GSE180904).

Downstream processing

Further processing of the dataset was performed with the python package Scanpy (Wolf et al. 2018). The dataset was subsequently log-transformed, scaled and using the HVGs as input a PCA reduction for the top 50 components was computed. A diffusion map embedding was also calculated for the top 15 diffusion components.

Differential expression

Differential expression on the Smart-seq2 sequencing data was performed using the R package DESeq2 (Love et al., 2014). The input expression matrices were filtered to include only genes with average expression above 1 (resulting in 14,080 genes on the filtered matrix). Differential expression results were selected for a significance level of 0.01. Visualization of results was done via volcano plot generated with the Enhanced Volcano R package (Blighe et al., 2018).

Gene set enrichment analysis (GSEA)

The Wald statistic from DESeq2 result was used to generate pre-ranked gene lists. These gene lists were then used for Gene set enrichment analysis (GSEA) with the GSEA software (v3.0) pre-ranked mode. Enrichment was tested for Hallmark and KEGG curated genesets of the MSigDB database v7.0 (Subramanian et al., 2005). Pathway enrichment was analyzed using Enrichr (https://maayanlab.cloud/Enrichr) and Gene Ontology was analyzed using Panther (http://www.pantherdb.org/).

STAT1KO and WT LK cells 10x Genomics data analysis

Bone marrow cells were harvested from STAT1KO and WT control mice and lineage negative, c-Kit^{+} (LK) cells were FACS sorted and processed according to the manufacturer's protocol for 10x Chromium (10x Genomics, Pleasanton, CA) experiments.

10x Preprocessing

The sequenced reads were processed with Cellranger (version 2.1.1) and aligned to the 10X Genomics built mouse mm10 reference (version 1.2.0). From the two libraries processed; a total of 13,770 cells were recovered. The raw sequencing reads and gene count tables were deposited at the NCBI GEO (accession number: GSE180905)

10x Downstream analysis

The remainder of the downstream analysis was performed using the Scanpy package. The Scrublet package was used to estimate doublets and 287 were identified and removed. The cells were further filtered based on percentage of mitochondrial UMI counts being less than 5% and cells expressing at least 500 genes. Genes were retained if they were expressed in at least in 3 cells. This resulted in a filtered dataset with 13,301 cells and 16,142 genes. The filtered dataset was subsequently normalized to the median UMI counts per cell across the two processed libraries and log-transformed. Highly variable genes were then computed with parameters *min_mean*=0.001, *max_mean*=5, *min_disp*=0.05 and the dataset scaled.

10x Dataset projection

This dataset was projected on a reference embedding representing LSK and LK populations (Dahlin et al. Blood. 2018) ("https://doi.org/10.1182/blood-2017-

12-821413"). Both our dataset and the reference were combined and the top 50 PCA components computed. The Euclidean distance in the PCA space between all cells in both datasets was then calculated. Our dataset consists of two samples (WT and KO) and for each the top k (100,000/size of sample) reference neighbors were considered for each of our cells. Within each sample every time a reference cell was identified as one of the k closest neighbors it incrementally grew the projection counter. This allowed us to create projection densities on the reference embedding. These projection counters were then smoothed by a factor of 1000 and used to calculate the log2-fold difference between each of the conditions which were then subsequently plotted on the reference embedding.

Visualizations

The embedding plots shown in this publication were generated through Scanpy's plotting methods. Violin plots were also generated using Scanpy or seaborn python packages. Heatmaps were generated with seaborn clustermap method using Euclidian distance metric with the 'ward' method for linkage.

Nestorowa Dataset (GSE81682):

The Smart-Seq2 HSPC dataset was obtained from Nestorowa et al. (Blood, 2016). The visualization was calculated using SPRING (Weinreb et al.,

Bioinformatics, 2017). Since the CMP population is heterogeneous, all CMP cells were reclassified into other cell types by first calculating Euclidean distance based on the top 50 PCA spaces and then looking for the 15 nearest neighbors for each CMP cell. The final assignment was based on the most frequent cell type within the nearest neighbors. In total, there are 249 LTHSCs in the dataset. The gene expression values indicated were calculated as log normalized counts. CD74 expression was plotted as a violin plot using the seaborn package in Python. There are 80 cells with CD74 expression > 5 and are labeled as CD74 high whereas there are 124 cells with CD74 expression < 3 and are labeled as CD74 low.

Differential pathway analysis between CD74 high and CD74 low LTHSCs were performed using the GO biological processes database (v7.1) downloaded from GSEA. Geometric means of genes in each term were calculated for all Nestorowa LTHSCs and t-test was applied using rank_genes_groups in Scanpy to test if means are the same between CD74 high cells and CD74 low cells. The significant terms were extracted from the MA plot with log2FC >= 0.5 and mean log2 expression >= -1.

Dahlin Dataset (GSE107727)

The 10x LK/LSK dataset was obtained from Dahlin et al. (Blood, 2018). The data was normalized to a total count of 10K and log transformed.

Mann dataset (GSE100426)

The HSC raw counts were downloaded from GEO and only unstimulated LT-HSCs from young aged mice were extracted for this study. The cells were filtered using filter_cells function in Scanpy with min_genes = 500 and the genes were filtered using the filter_genes function with min_cells=1. Then the data was normalized to a total count of 10K and logarithmized. In total, there are 88 young LTHSCs.

Haltallli dataset GSE156410

Four Plasmodium infected samples (Haltallli et al.) GSE156410 were downloaded from GEO database as filtered cellranger output h5 files, representing 2 experimental groups: infected and control, with 2 biological replicates each. Preprocessing was done using the Scanpy pipeline. Each cell was normalized to 10K for comparison and logged. Cell cycle scores were calculated using the score_genes_cell_cycle function. High variable genes were selected from each sample using highly_variable_genes, with min_mean=0.02, max_mean=3, min_disp=0.3 and merged. In total, 1612 genes were considered as highly variable. In order to capture cell type differences as the most dominant component, cell cycle phases, number of genes, number of counts and percentage of mitochondrial genes were regressed out. Four samples were further integrated using reducedMNN function in batchelor R package. Cell types assignment was done using the

Nestorowa landscape for the HSC and immature populations and Dahlin landscape for the mature populations as references. Cells were clustered using Leiden clustering with resolution=1. LT-HSCs were extracted with 2 criteria: 1) Cell type was defined as LT-HSC; 2) Leiden cluster = 1 that contains HSC and immature populations to further exclude the non-specific cells.

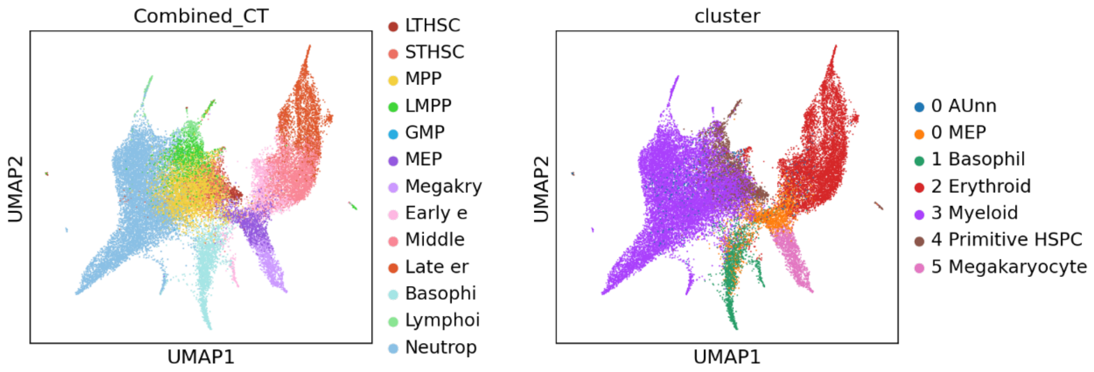

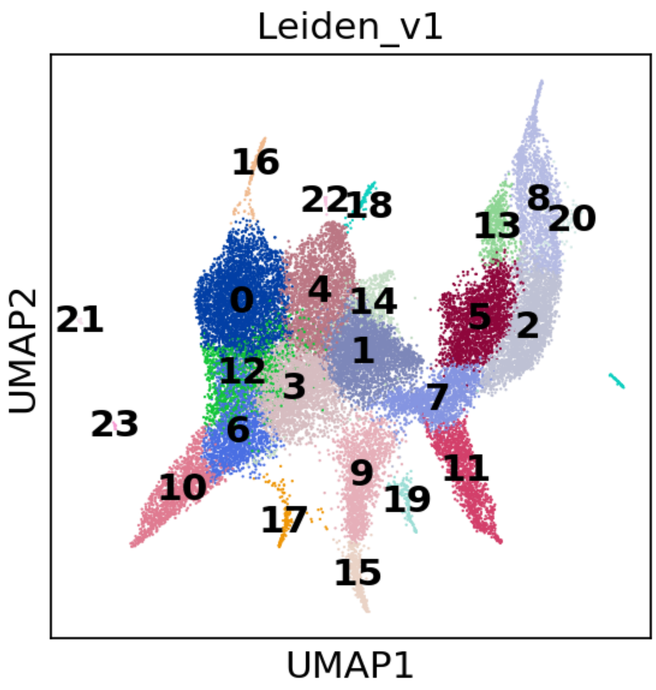

Violin plots were done using ggplot2 package in R. Means difference between infected and control were tested using t test.

	GeneName	.y.	group1	group2	n1	n2	statistic	df	p	p.signif
1	H2-Eb1	log_Exp	Control	Infected	181	19	-18.3156	22.61219	4.68E-15	****
2	H2-Aa	log_Exp	Control	Infected	181	19	-25.533	33.73149	1.21E-23	****
3	H2-Ab1	log_Exp	Control	Infected	181	19	-25.9933	33.34646	1.03E-23	****
4	Cd74	log_Exp	Control	Infected	181	19	-28.9546	44.14033	2.38E-30	****
5	Ciita	log_Exp	Control	Infected	181	19	-1.37449	18.20944	1.86E-01	ns
6	Stat1	log_Exp	Control	Infected	181	19	-10.2442	21.56432	9.60E-10	****

CALR 10x scRNAseq dataset (GSE160466)

Firstly, CALR cells were projected onto the Dahlin landscape by a PCA projection. Euclidean distances were calculated between CALR cells and the Dahlin data based on the top 50 PCA components. Top 15 nearest neighbor cells from the Dahlin landscape were calculated for each CALR cell with the shortest Euclidean distance. The cell type annotation was then assigned as the most frequent cell type from the nearest neighbors. To annotate HSPCs in more details, the HSCs and immature populations of the CALR dataset were extracted and projected again onto the Nestorowa landscape with the same procedure indicated above. Finally, LTHSCs were extracted for this study. 669 and 506 LTHSCs were extracted from the WT CALR mutant datasets respectively. Then the data was normalized to a total count of 10K and log transformed.

Score calculation

G2M, S and MHC scores were calculated using score_genes function in Scanpy. G2M and S gene lists were defined in Tirosh et al, 2015. MHC genes used are Cd74, H2-Aa, H2-Ab1 and H2-Eb1. The data was log-normalized, and

scaled before applying the score calculation. All plots were done using the Python seaborn package.

References

Anders, S., Pyl, P.T., and Huber, W. (2015). HTSeq—a Python framework to work with high-throughput sequencing data. 31, 166–169.

Blighe, K, S Rana, and M Lewis. 2018. “EnhancedVolcano: Publication-ready volcano plots with enhanced colouring and labeling.” ("https://github.com/kevinblighe/EnhancedVolcano.")

Brennecke, P., Anders, S., Kim, J.K., Kołodziejczyk, A.A., Zhang, X., Proserpio, V., Baying, B., Benes, V., Teichmann, S.A., Marioni, J.C., et al. (2013). Accounting for technical noise in single-cell RNA-seq experiments. 10, 1093–1095.

Durbin, J.E., Hackenmiller, R., Simon, M.C., and Levy, D.E. (1996). Targeted disruption of the mouse Stat1 gene results in compromised innate immunity to viral disease. Cell 84, 443-450.

Li, J., Prins, D., Park, H.J., Grinfeld, J., Gonzalez-Arias, C., Loughran, S., Dovey, O.M., Klampfl, T., Bennett, C., Hamilton, T.L., et al. (2018). Mutant calreticulin knockin mice develop thrombocytosis and myelofibrosis without a stem cell self-renewal advantage. Blood 131, 649-661.

Love, M.I., Huber, W., and Anders, S. (2014). Moderated estimation of fold change and dispersion for RNA-seq data with DESeq2. 15

Prins, D., Park, H.J., Watcham, S., Li, J., Vacca, M., Bastos, H.P., Gerbaulet, A., Vidal-Puig, A., Gottgens, B., and Green, A.R. (2020). The stem/progenitor

landscape is reshaped in a mouse model of essential thrombocythemia and causes excess megakaryocyte production. Science advances 6 (48).

Ritchie ME, Phipson B, Wu D, Hu Y, Law CW, Shi W, Smyth GK (2015). “limma powers differential expression analyses for RNA-sequencing and microarray studies.” Nucleic Acids Research, 43(7), e47. doi: "https://doi.org/10.1093/nar/gkv007".

Sanjuan-Pla, A., Macaulay, I.C., Jensen, C.T., Woll, P.S., Luis, T.C., Mead, A., Moore, S., Carella, C., Matsuoka, S., Bouriez Jones, T., et al. (2013). Platelet-biased stem cells reside at the apex of the haematopoietic stem-cell hierarchy. Nature 502, 232-236.

Subramanian, A., Tamayo, P., Mootha, V.K., Mukherjee, S., Ebert, B.L., Gillette, M.A., Paulovich, A., Pomeroy, S.L., Golub, T.R., Lander, E.S., et al. (2005). Gene set enrichment analysis: A knowledge-based approach for interpreting genome-wide expression profiles. 102, 15545–15550.

Wolf, F., Angerer, P. & Theis, F. SCANPY: large-scale single-cell gene expression data analysis. (2018) Genome Biol 19, 15 https://doi.org/10.1186/s13059-017-1382-0

Wu, T.D., and Nacu, S. (2010). Fast and SNP-tolerant detection of complex variants and splicing in short reads. 26, 873–881

Zerbino, D.R., Achuthan, P., Akanni, W., Amode, M.R., Barrell, D., Bhai, J., Billis, K., Cummins, C., Gall, A., Girón, C.G., et al. (2018). Ensembl 2018. 46, D754–D761.

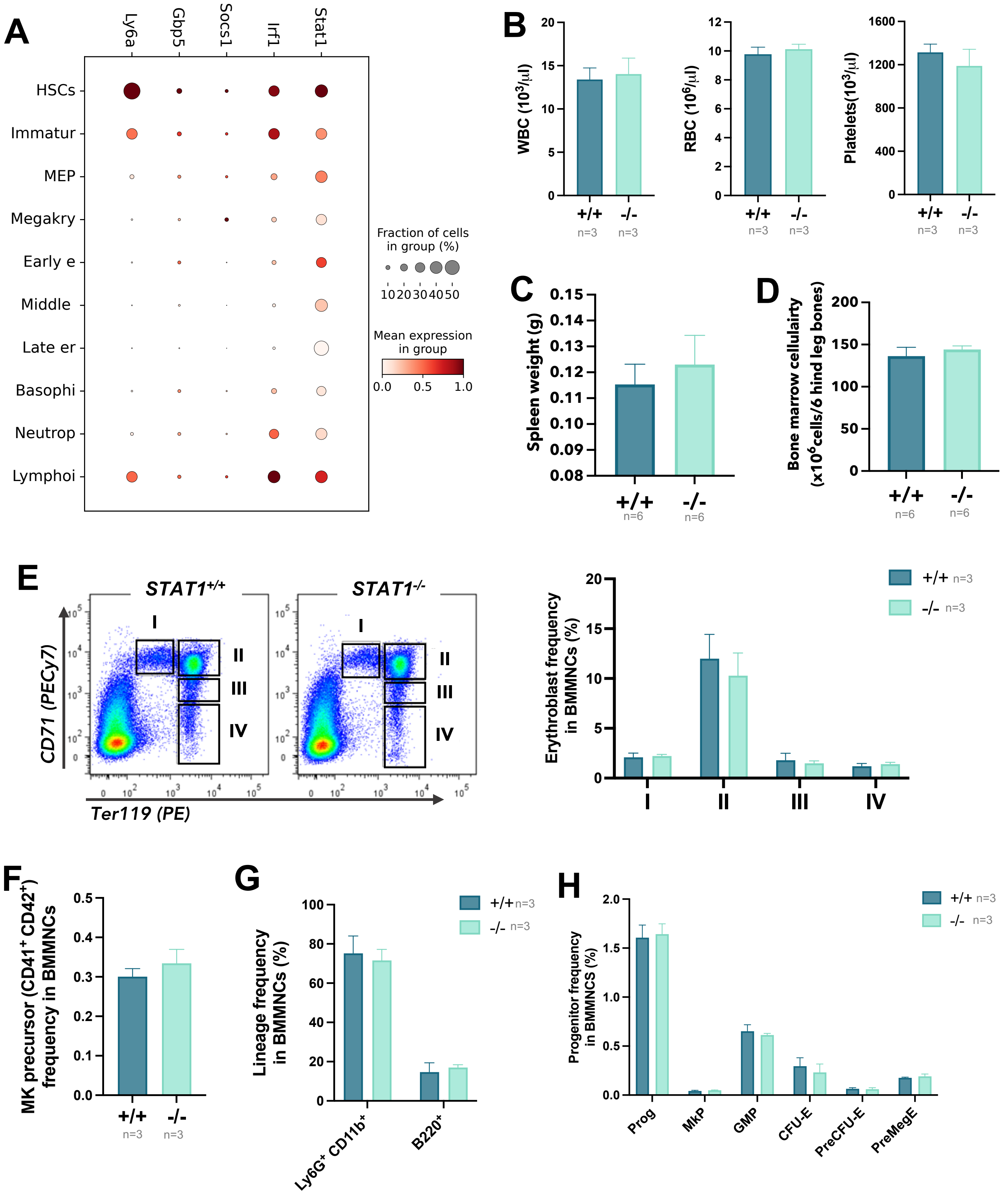

Supplemental Figure 1

Figure S1: STAT1-deficient mice display normal blood phenotypes and mature lineage cell frequencies in bone marrow

(A) Dot plot showing normalized expression of pSTAT1 proto-typical target genes in cell types across the Dahlin landscape. The size of each dot indicates the proportion of cells with normalized expression level >0 and the color intensity shows the levels of gene expression.

(B) STAT1KO mice show normal peripheral blood phenotypes.

(C) STAT1KO mice show normal spleen sizes.

(D) STAT1KO mice show normal bone marrow cellularity.

(E) Representative plots of erythroblast gating (Left panel), bar graph showing the frequencies of erythroblasts at different stages of terminal differentiation in bone marrow (Right panel).

(F) Bar graph showing the frequencies of megakaryocytes (MK) in bone marrow.

(G) Frequency of myeloid and lymphoid lineage cells in bone marrow.

(H) Frequency of progenitors in bone marrow.

Data are shown as mean $\pm$ SEM.

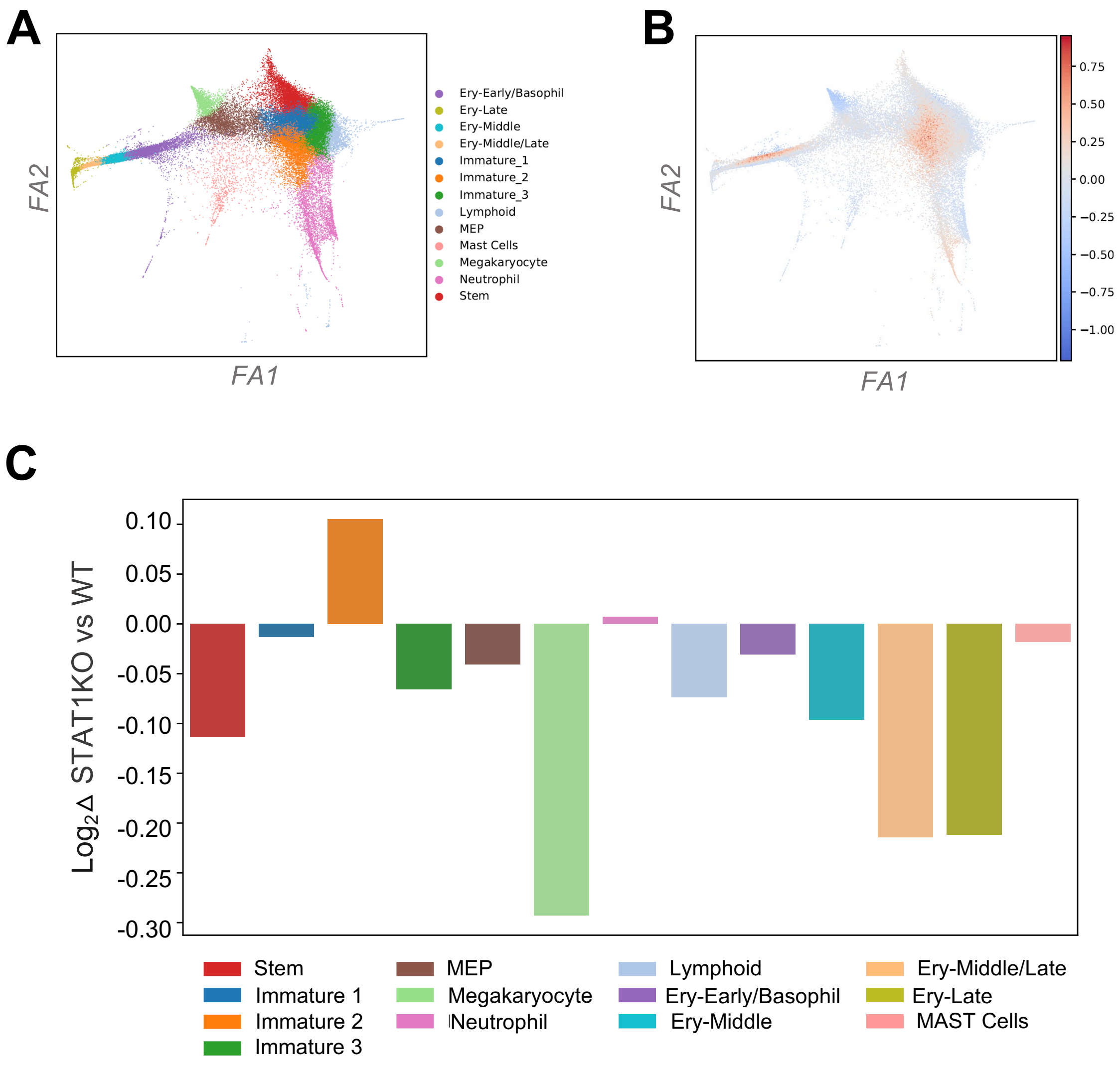

Supplemental Figure 2

Figure S2: Loss of STAT1 affects transcriptomic landscape of HSPCs

(A) Force-directed graph from Dahlin et. al., (2018) showing the landscape of LK and LSK cells with annotated clusters indicated.

(B) Projection of scRNAseq data from LK cells isolated from STAT1KO and WT control mice onto the force-directed graph from (A). Red and blue colors indicate relative enrichment or depletion respectively in STAT1KO cells.

(C) Bar graph showing the relative abundance of each cell type.

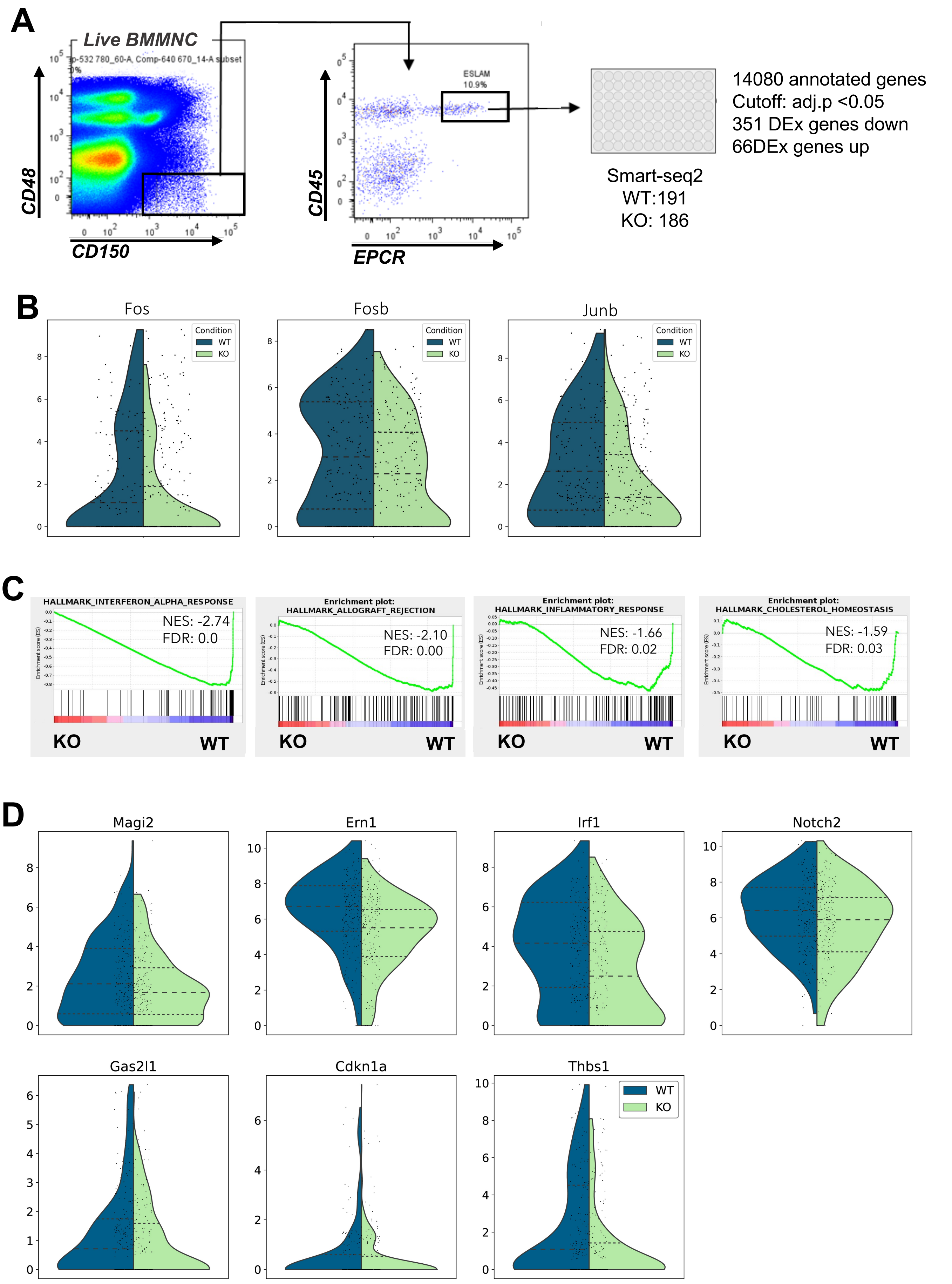

Supplemental Figure 3

Figure S3: scRNAseq reveals enrichment of cell cycle related signatures in STAT1-deficient ESLAM HSCs

(A) Experimental scheme of the ESLAM HSC scRNAseq analysis.

(B) Violin plots showing the normalized expression of genes encoding transcription factors of the AP-1 family.

(C) GSEA plots showing significantly depleted signatures in STAT1-deficient ESLAM HSCs.

(D) Violin plots showing the normalized expression of genes related to cell cycle arrest.

A

D14	WBC	RBC	HGB	HCT	MCV	MCH	MCHC	PLT
**Stat1 +/+**	23.6	9.4	10.5	42.9	46	11.2	24.5	4126
**Stat1 +/+**	16.6	9.72	10.9	45.8	47	11.2	23.8	2298
**Stat1 +/+**	14.3	9.67	11.2	45.9	47	11.6	24.3	1868
**Stat1 -/-**	16	8.16	9.6	38.2	47	11.8	25.2	3642
**Stat1 -/-**	9.9	9.69	11.4	47.6	49	11.7	23.9	808
**Stat1 -/-**	11.8	10.15	11.7	49.4	49	11.5	23.7	606

B

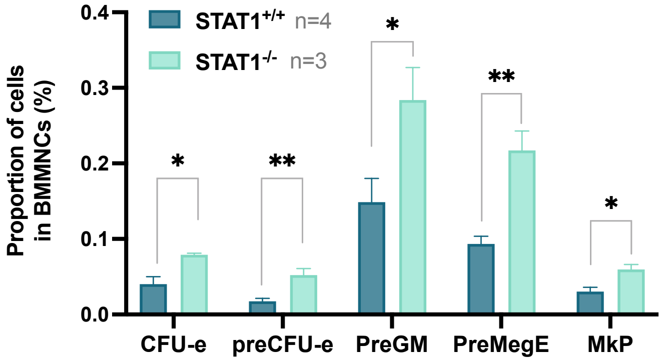

C

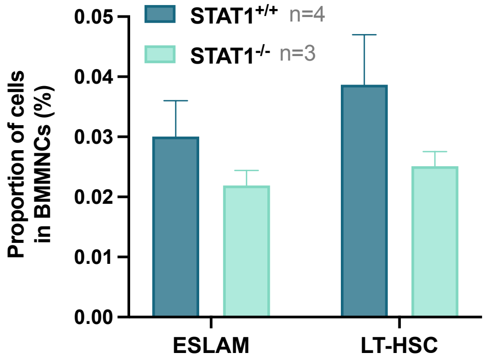

Supplemental Figure 4

Figure S4: STAT1-deficient mice display delayed blood rebound following 5'-FU induced myeloablation

(A) STAT1-deficient mice showed delayed rebound of platelets and WBC during recovery of blood regeneration following 5-FU treatment; blood counts at day 14 are shown.

(B) Flow cytometric analysis showing the frequency of myeloid progenitors in BMMNCs at day 15 following 5-FU treatment. Mean ± SEM; **, $p<0.01$; *, $p<0.05$.

(C) Frequency of ESLAM HSCs was not significantly different in STAT1-deficient mice at day 15 following 5-FU treatment.

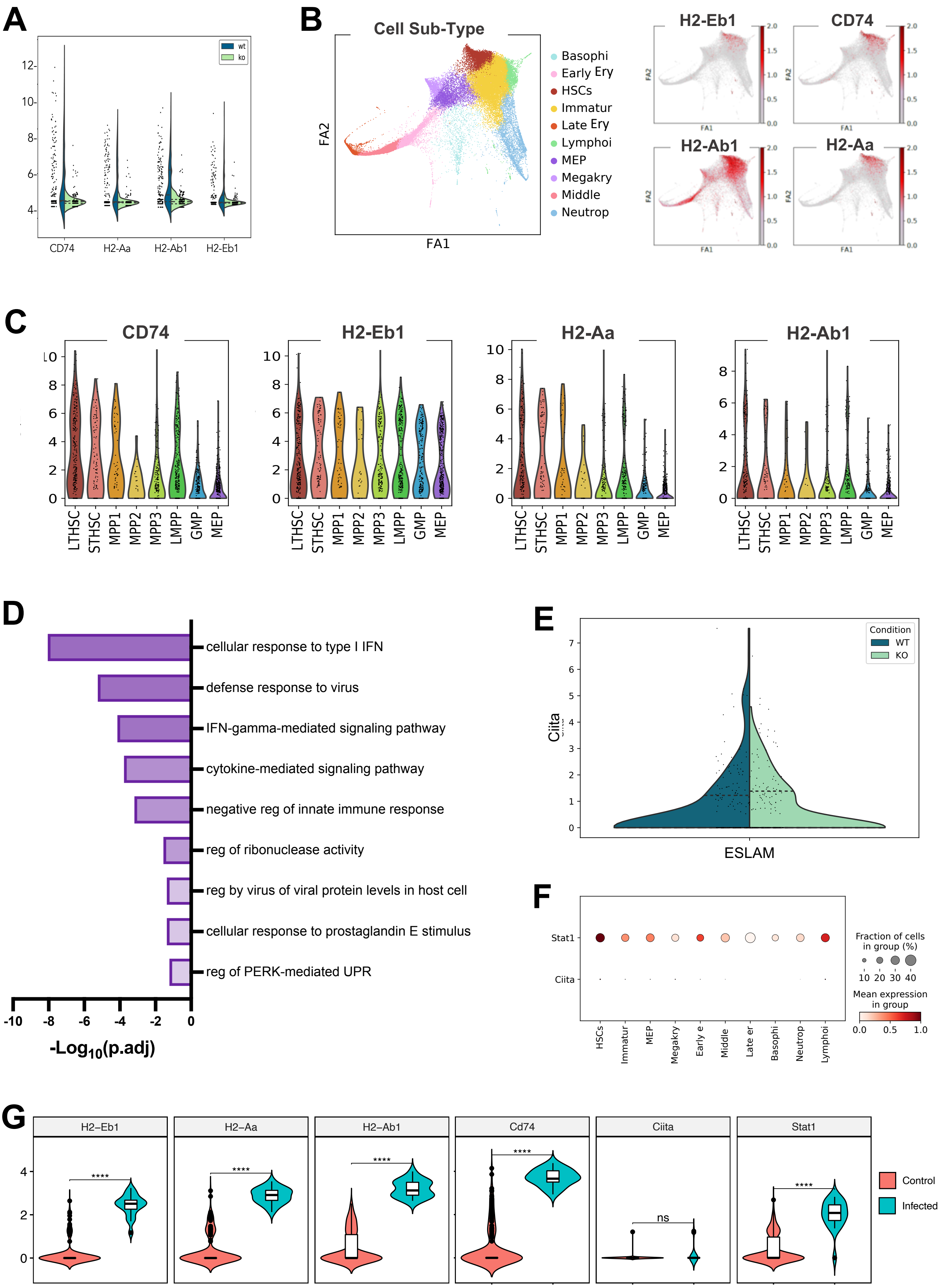

Supplemental Figure 5

Figure S5: STAT1 is required to maintain a subset of HSCs expressing high level of MHCII

(A) Violin plots showing normalized MHCII gene expression in homeostatic ESLAM HSCs at steady state.

(B) Force directed graphs showing the expression abundance of MHCII genes within LK and LSK cells from the Dahlin (2018) dataset.

(C) Violin plots showing normalized MHCII gene expression in immature cell types in the Nestorowa (2015) dataset.

(D) GO enrichment analysis showing down-regulated terms in STAT1-deficient ESLAM HSCs compared to WT HSCs excluding MHCII^{hi} cells. Statistical significance is indicated by -Log10 (p.adj).

(E) Violin plot showing *Ciita* expression is not significantly down regulated in STAT1KO HSCs.

(F) Dot plot showing normalized *Ciita* and *Stat1* expression in cell types across the Dahlin landscape. The size of each dot indicates the proportion of cells with normalised expression level >0 and the colour intensity shows the levels of gene expression.

(G) Violin plots showing gene expression in LT-HSCs from untreated mice and mice infected with plasmodium (dataset of Haltalli et al 2020).

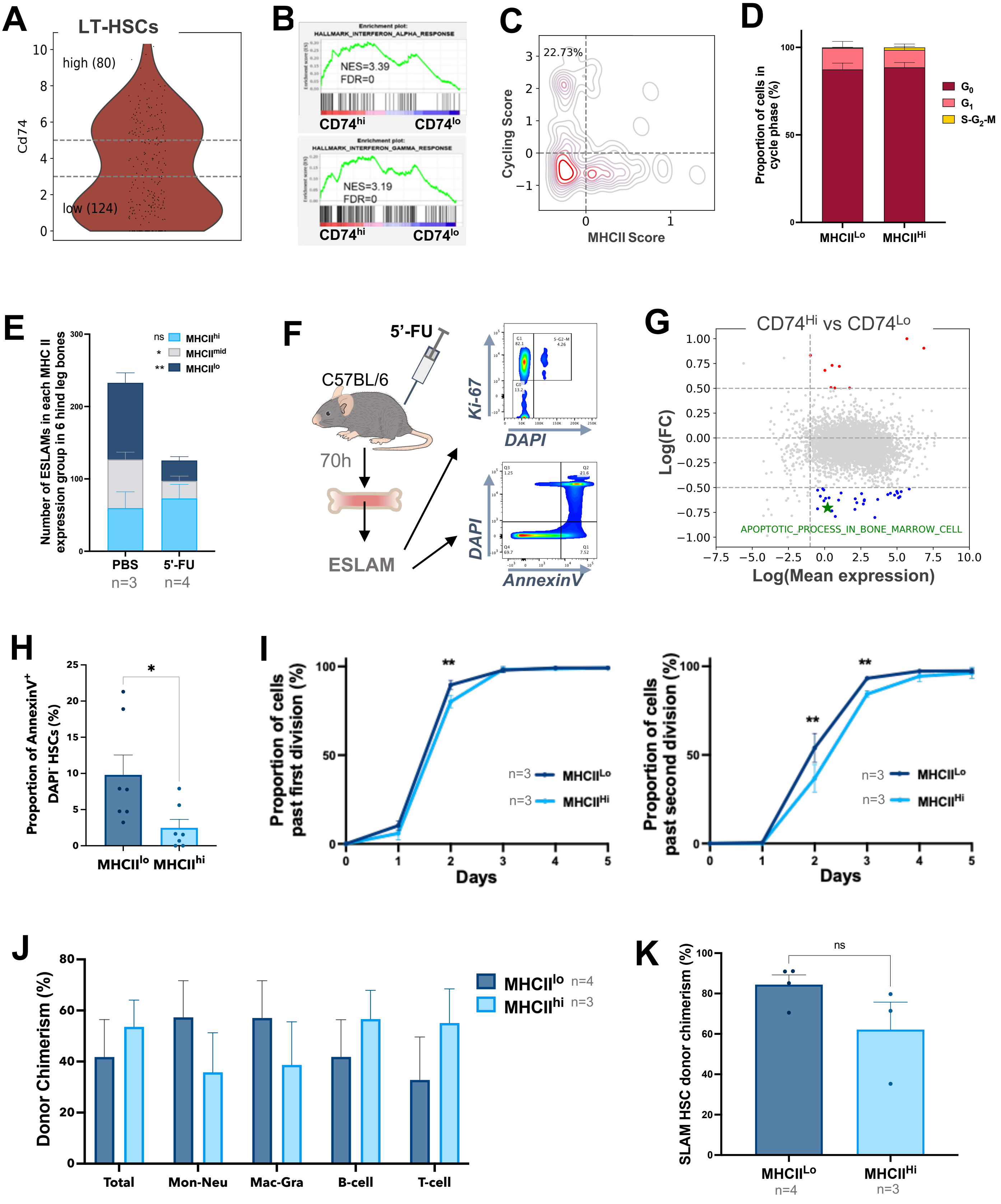

Supplemental Figure 6

Figure S6: HSCs expressing high levels of MHCII display distinct functionality

(A) Violin plot showing CD74 expression in LTHSCs in the Nestorowa dataset with indicated CD74^{hi} and CD74^{lo} fractions.

(B) GSEA plots showing the enrichment of response to IFN signatures in CD74^{hi} LTHSCs.

(C) LTHSCs with low MHCII expression scores in de Boer dataset (GSE100426, young LTHSCs) tend to display higher cycling scores.

(D) Cell cycle analysis using Ki-67/DAPI staining, bar graphs showing similar cell cycle status frequency between MHCII^{hi} and MHCII^{lo} ESLAM HSCs at steady state.

(E) Bar graphs showing the number of HSCs in low and mid MHCII expressing subsets are depleted following 5-FU induced myeloablation.

(F) Experimental scheme for analyzing ESLAM HSC cell cycle and apoptosis following 5-FU treatment.

(G) MA plot showing enrichment analysis of GO pathways. Red circles indicate upregulated and blue indicated down-regulated pathways respectively. Apoptosis is indicated with a green star.

(H) Apoptosis analysis using Annexin V/DAPI staining, bar graphs showing a lower rate of apoptosis in MHCII^{hi} ESLAM HSCs compared to MHCII^{lo} HSCs at steady state.

(I) Line graphs showing significantly lower proportion of E-SLAM HSCs completed first division and second during the 5 days of single cell in vitro analysis. **, $P<0.01$; p-value was calculated from a chi-squared test; three independent experiments were performed.

(J) Bar graphs showing donor chimerisms in peripheral blood at 16 weeks post transplantation of MHCI^{lhi} and MHCII^{lo} ESLAM HSCs. Total, CD45.2^{+} ; Mon-Neu, Ly6G^{+} ; Mac-Gra, CD11b^{+} ; B-cell, B220^{+} ; T-cell, CD3e^{+}.

(K) Bar graphs showing donor derived HSC chimerisms in MHCII^{hi} primary recipients. Recipient bone marrows were analyzed 16 weeks post transplantation.

Data are shown as mean $\pm$ SEM; asterisks indicate significant differences by Student's t test (**, $p<0.01$; *, $p<0.05$).

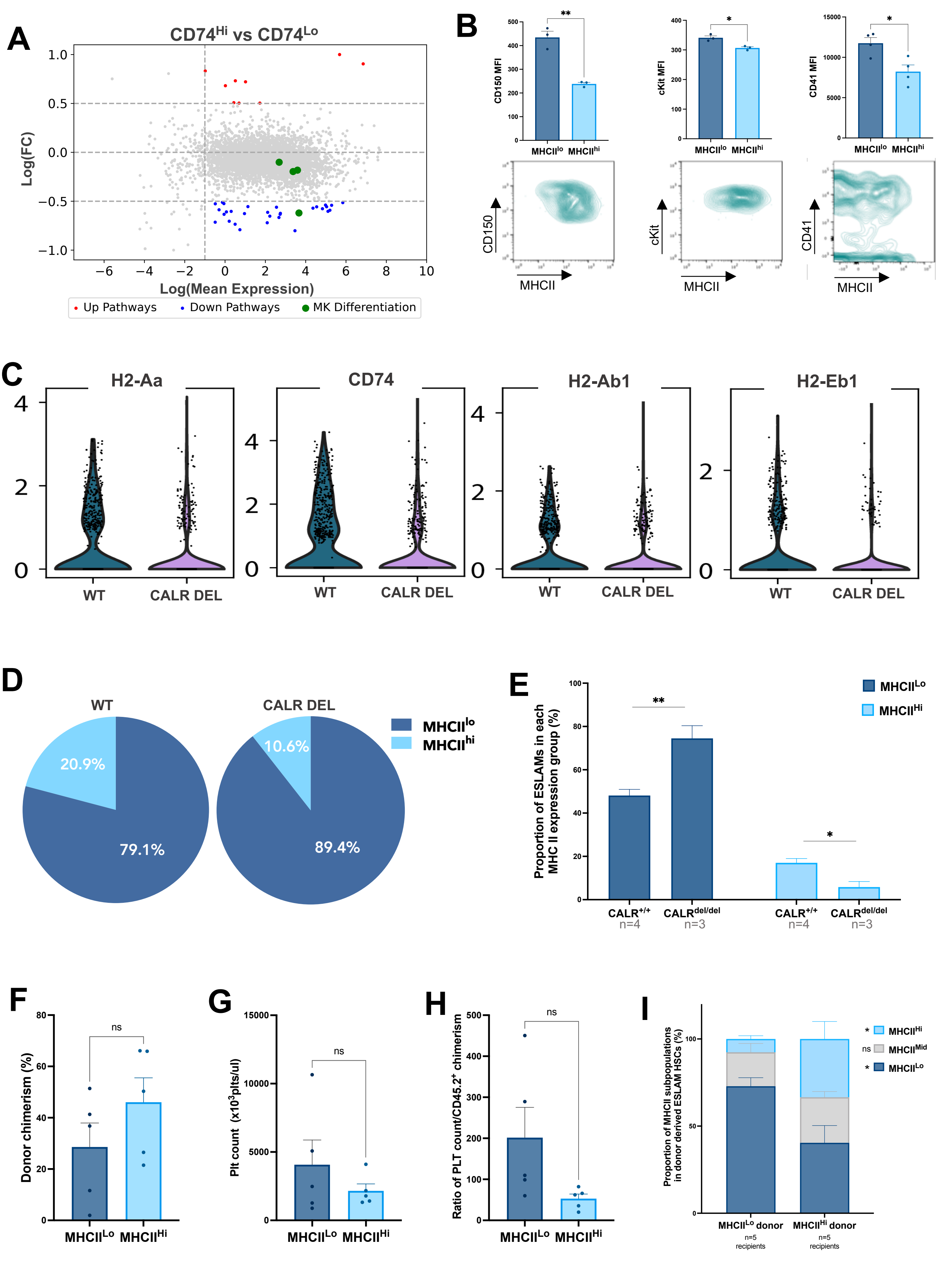

Supplemental Figure 7

Figure S7: $MHCII^{lo}$ HSCs exhibit enhanced megakaryocytic differentiation and are preferentially expanded in mutant CALR mice with thrombocythemia/ mylofibrosis

(A) MA plot showing enrichment analysis of GO pathways. Red and blue circles indicate up-regulated and down-regulated pathways in $CD74^{hi}$ LTHSCs, respectively. Differentially regulated pathways for megakaryocytic differentiation are labelled with a green star.

(B) Bar graphs showing MFI levels of respective cell surface marker as indicated; representive flow cytometry plots are shown.

(C) Violin plots showing reduced expression of MHCII genes in LTHSCs from mutant CALR knock-in mice

(D) Pie charts showing the increased proportion of transcriptionally defined $MHCII^{lo}$ LTHSCs from CALR mutant mice compared to WT control.

(E) Bar graphs showing higher proportion of $MHCII^{lo}$ ESLAM HSCs in CALR mutant BMMNCs assessed by flow cytometric analysis.

(F) Bar graphs showing 45.2 chimerisms in peripheral bloods of primary recipients at 16 weeks post transplantation.

(G) Bar graphs showing platelet counts in recipient mice of CALR mutant $MHCII^{hi}$ or $MHCII^{lo}$ ESLAM HSCs at 16 weeks post transplantation.

(H) Bar graphs showing ratios of platelet counts / 45.2 chimerisms in recipient mice of CALR mutant $MHCII^{hi}$ or $MHCII^{lo}$ ESLAM HSCs at 16 weeks post transplantation.

(I) Bar graphs showing proportions of ESLAM HSC subpopulations (based on MHCII levels; $MHCII^{hi}$, $MHCII^{lo}$ and $MHCII^{mid}$) in recipient mice of CALR mutant $MHCII^{hi}$ or $MHCII^{lo}$ HSCs at 16 weeks post transplantation.

Data are shown as mean ± SEM; asterisks indicate significant differences by Student's t test unless indicated (**, $p<0.01$; *, $p<0.05$).
